# Use of the patient-reported outcomes measurement information system (PROMIS®) to assess late-onset Pompe disease severity

**DOI:** 10.1186/s41687-020-00245-2

**Published:** 2020-10-09

**Authors:** Melodi Harfouche, Priya S. Kishnani, Eva Krusinska, Jamie Gault, Sheela Sitaraman, Amanda Sowinski, Irina Katz, Stephanie Austin, Margi Goldstein, Andrew E. Mulberg

**Affiliations:** 1 Amicus Therapeutics, Inc., 1 Cedar Brook Drive, Cranbury, NJ 08512 USA; 2 Duke University Medical Center, 905 South Lasalle Street GSRB1 Building, 4th Floor, Room 4010, Durham, NC 27710 USA; 3 Formerly Amicus Therapeutics; currently Andrew E. Mulberg, MD, LLC, 52 Saint Moritz Lane, Cherry Hill, New Jersey 08003 USA

**Keywords:** PROMIS, Pompe disease, Patient-reported outcomes (PROs), Clinical research, Drug development

## Abstract

**Background:**

Patient-Reported Outcomes provide an opportunity for patients to establish dialogue with pharmaceutical or biotechnology companies about their health conditions without interpretation by a clinician or anyone else. However, Patient-Reported Outcomes that can be widely applicable for use in patient-focused drug development or clinical trial designs are not yet validated for all diseases. The aim of this study report was to provide supportive evidence of the construct and content validity of selected Patient-Reported Outcomes Measurement Information System (PROMIS®) questionnaires compared with other disease-relevant clinical outcome measures, including the 6-Minute Walk Distance, forced vital capacity, and Manual Muscle Test, in late-onset Pompe disease and to provide supportive evidence that the selected PROMIS measures are relevant and important to these patients.

**Methods:**

Thirty patients with late-onset Pompe disease completed five PROMIS questionnaires that were chosen based on patient and provider feedback, along with discussion with key opinion leaders who are experts in Pompe disease. The Amicus Pompe Patient Advisory Board also provided patient experience feedback using the PROMIS questionnaires. Clinical outcome measures (6-Minute Walk Distance, forced vital capacity, and Manual Muscle Test) were collected at the Duke University Pompe Disease Clinical Research Program during a single visit.

**Results:**

The Patient Advisory Board rated the questionnaires as representative of an unmet need. Correlation data demonstrated moderate to strong correlations of PROMIS questionnaires with the specified clinical outcome measures (6-Minute Walk Distance, forced vital capacity, and Manual Muscle Test). These data supported the construct and content validity of the PROMIS questionnaires because they confirmed the motor signs and symptoms of functional disability observed in patients with Pompe disease.

**Conclusions:**

The correlations indicate that the clinical outcome measures assess important concepts related to patient-reported experiences. The Patient Advisory Board findings suggest that the selected PROMIS questionnaires are meaningful and address important concepts to patients with Pompe disease. The data were collected from a small number of patients at a single time point; further studies are needed with additional PROMIS questionnaires, which should include measures of motor function and health-related quality of life, in a larger number of patients followed up longitudinally.

## Background

### Introduction

Pompe disease (OMIM #232300, also known as acid maltase deficiency or glycogen storage disease type II) is a rare autosomal recessive genetic disorder caused by pathogenic variants in the gene that encodes acid α-glucosidase (GAA). These variants result in complete absence or partial loss of endogenous GAA activity, leading to accumulation of lysosomal glycogen and progressive disruption of cellular function, particularly in cardiac, skeletal and smooth muscles, and the diaphragm.

Pompe disease is a serious, progressive, debilitating, and ultimately life-threatening disease associated with high morbidity and is categorized into two classes: infantile-onset Pompe disease (IPD) and late-onset Pompe disease (LOPD) [1].

LOPD encompasses childhood, juvenile, and adult-onset disease, with variable severity manifesting from infancy to the sixth decade of life. Proximal lower limb and paraspinal trunk muscles usually are affected first, followed by further involvement of skeletal muscles and respiratory muscles, particularly the diaphragm and the intercostal and accessory muscles. In some cases, diaphragmatic weakness may be evident before any other significant weakness is noted [2]. Although significant clinical benefits have been attained with the standard of care enzyme replacement therapy alglucosidase alfa (Myozyme® [EMEA/H/C/000636]/Lumizyme®; Biologics License Applications 125,291, approved 24 May 2010), there typically is a clinical plateau or a decline over time. Most patients with LOPD eventually progress to physical debilitation requiring the use of a wheelchair and assisted ventilation, with premature death often occurring due to respiratory failure [2, 3].

As drug licensing in the United States transitions toward a more patient-centered approach to identifying a clinically meaningful definition of endpoint, patient-reported outcomes (PROs) provide an opportunity for patients to establish dialogue with pharmaceutical or biotechnology companies. According to the US Food and Drug Administration (FDA), a PRO is any report of the status of a patient’s health condition that comes directly from the patient, without interpretation of the patient’s response by a clinician or anyone else. PROs can assess the effects of a specific clinical intervention that may or may not be physically observable and can provide insight to the patient’s perceptions about how the disease or treatment is affecting their health-related quality of life (HRQoL) [4]. This outcome can be measured in terms of severity of a symptom, sign, or state of disease or as a change from a previous measure [5].

Chronic illnesses have a large impact on HRQoL within the three domains of physical, psychological, and social functioning [6]. Symptoms such as fatigue, pain, and muscle weakness and atrophy can affect everyday activities of daily living (ADL), leading to an overall effect on HRQoL. A previous study on pain in patients with Pompe disease found that pain is related to reduced HRQoL, less participation in daily life, and greater depression and anxiety [7].

Medical technology can measure physical, physiological, and biochemical data about a patient, yet it is unable to provide all the unobservable data regarding a treatment or a disease [4]. Improving HRQoL and reducing the burden of disease play a major role in patient satisfaction, yet in the setting of a clinical trial this is often not captured. In chronic diseases for which economical strain and overall burden of disease can adversely affect a patient’s life, PROs can help assess the HRQoL in such patients. The adjunctive use of endpoints based on PROs, in conjunction with quantitative clinical assessments, may provide a substantial body of evidence to support the conclusion that a treatment or a drug is providing clinical benefit.

Several different PRO instruments are available to assess the burden of a disease and the impact on a patient’s physical, psychological, and social well-being [4]. One such set of instruments is the Patient-Reported Outcomes Measurement Information System, or PROMIS® [8]. PROMIS provides patient-reported or observer-based outcome assessments to assess various PROs such as pain, fatigue, physical functioning, emotional distress, and social well-being, all of which play a significant role in HRQoL assessments. PROMIS tools administered to patients investigate not only the level of physical impact but also the burden of disease on psychological and social well-being through the use of questionnaires, item banks, or otherwise. Collecting input from people living with a disease is critical for ensuring adequate and efficient identification of clinically meaningful endpoints to improve the clinical meaningfulness of individual drug development programs.

The PROMIS domains herein reported and used reflect clinically relevant signs and symptoms of Pompe disease, including respiratory and motor signs and symptoms (eg, fatigue). The five PRO assessments included in this study were selected PROMIS questionnaires that assessed signs and symptoms known to be associated with Pompe disease (pain interference, upper extremity function, fatigue, physical function, and dyspnea). In one study, fatigue was found to be highly prevalent among both mildly and severely affected adult patients with Pompe disease using the Fatigue Severity Scale [9]. Another observational study in patients with Pompe disease noted that fatigue and muscle cramps were among the first symptoms they experienced [3]. Muscle weakness was most often mentioned in regard to other signs and symptoms (80%), namely difficulty walking, delayed motor development, and hypotonia [10]. Respiratory muscle weakness is also characteristic of LOPD, with approximately 75% of children and adolescents eventually needing mechanical ventilation [11]. In one study that assessed pain severity in patients with Pompe disease, it was noted that pain also interferes with general activities such as walking, work, mood, sleep, and enjoyment of life. Of 124 patients in the study, 45% reported having pain [7].

The Duke University Pompe Disease Clinical Research Program in collaboration with Amicus Therapeutics (Amicus) supported a study in patients with LOPD to select PROs that best capture Pompe symptomology and assess how well-selected PROMIS questionnaires correlate with clinical assessment by a physical therapist. Other disease-relevant clinical outcome measures, including the 6-Minute Walk Distance (6MWD), forced vital capacity (FVC), and Manual Muscle Test (MMT). In parallel, the Amicus Pompe Patient Advisory Board (PAB; a group of informed individuals living with Pompe disease or those who care for individuals with Pompe disease) was asked to evaluate the **construct and content validity** of the selected PROMIS questionnaires and the impact of Pompe disease on HRQoL.

### Purpose

The purpose of this report is to provide evidence of the **construct and content validity** of selected PROMIS questionnaires relative to the other disease-relevant clinical outcome measures, including the 6MWD, FVC, and MMT, in patients with LOPD and to provide evidence that the selected PROMIS questionnaires are relevant and important to patients with LOPD.

## Methods

### Selection of subjects

The Pompe long-term natural history study was approved by the Duke University Institutional Review Board (IRB, Pro00083673). The study involved a single-center, single-arm study in adult patients with LOPD conducted at the Duke University Pompe Disease Program. Patient eligibility criteria included age 18 years or older, confirmed diagnosis of LOPD by molecular or enzymatic testing, and care by a clinical geneticist with expertise in Pompe disease (PSK). There were no interventions or interactions with the patients by Amicus as part of this study.

Data were collected from qualifying patients at a single point in time during a regularly scheduled clinical appointment. Within the same visit, patients completed five PROMIS questionnaires **in format of paper measurement** and underwent assessment (6MWD, FVC, and MMT) by a physical therapist with expertise in neuromuscular disorders. Clinical data that were collected also included each patient’s age, sex, weight/body mass index, and *GAA* genotype. Data were then entered into the Research Electronic Data Capture database, a web-based application intended to collect data for research.

The five PROMIS questionnaires were Pain Interference Short Form (SF) 8a, Upper Extremity SF 7a, Fatigue SF 8a, Physical Function SF 20a, and Dyspnea SF 10a [8]. They varied in the number of questions asked and their rating scale. Among the many different PROMIS questionnaires available, the five chosen for this study are considered clinically relevant to patients with LOPD based on patient and provider feedback from the PAB, review of the literature, and discussion with key opinion leaders who are experts in Pompe disease. Table 1 lists the five PROMIS questionnaires the patients completed as well as the number of questions on each questionnaire, the score range, and the definition of each PROMIS score.

**Table 1:** PROMIS Questionnaires Administered

PROMIS Questionnaire	Number of Questions	Minimum Score for Each Item	Maximum Score for Each Item	Meaning
Pain Interference Short Form 8a	8	1 “not at all”	5 “very much”	The lower the score, the less pain was an interference in ADL and HRQoL
Upper Extremity Short Form 7a	7	1 “unable to do”	5 “without any difficulty”	The higher the score, the more mobility with upper extremities
Fatigue Short Form 8a	8	1 “not at all”	5 “very much”	The lower the score, the less impact fatigue had on ADL and HRQoL
Physical Function Short Form 20a	20	1 “unable to do”	5 “without any difficulty”	The higher the score, the less impact on physical function
Dyspnea Short Form 10a	10	0 “no shortness of breath”	3 “severely short of breath”	The lower the score, the less limitation by breathlessness

*ADL* Activities of daily living, *HRQoL* Health-related quality of life, *PROMIS* Patient-Reported Outcomes Measurement Information System

Three clinical outcome measures—6MWD (in meters and % predicted), MMT (overall, upper, and lower), and FVC (upright, supine, and % predicted)—were assessed for correlation with each of the five chosen PROMIS questionnaires (Pain Interference SF 8a, Upper Extremity SF 7a, Fatigue SF 8a, Physical Function SF 20a, Dyspnea SF 10a). A correlation was expected between 6MWD and Physical Function SF 20a because an increase in physical limitation would result in a shorter distance walked in 6 min. A correlation was expected between FVC and Dyspnea SF 10a because shortness of breath would result in lower forced expiration. However, correlation scores were calculated for each clinical outcome measure against each PROMIS questionnaire to identify any correlations that were not originally expected (Table 2).

**Table 2:** Correlations Between PROMIS Questionnaires and Clinical Outcome Assessments

6MWD (meters and % predicted) MMT (overall, upper extremities, and lower extremities) FVC (upright, supine, and % predicted)	Pain Interference SF 8a Upper Extremity SF 7a Fatigue SF 8a Physical Function SF 20a Dyspnea SF 10a

*6MWD* 6-Minute Walk Distance, *FVC* Forced vital capacity, *MMT* Manual Muscle Test, *PROMIS* Patient-Reported Outcomes Measurement Information System, *SF* Short Form

A low correlation is any correlation score of ≤0.4. A moderate correlation is observed with a correlation score of ≥0.5 to < 0.7. A strong correlation is any correlation score of ≥0.7.

Correlation scores were also measured within each PROMIS questionnaire against each clinical outcome assessment. The individual questions in all five PROMIS questionnaires were correlated against each clinical outcome measure to assess whether any question caused skewing of the correlation score of the overall PROMIS questionnaire against each clinical outcome assessment. 6MWD was chosen as the relevant clinical outcome based on FDA acceptance of this measurement to assess clinical benefit and on FDA approval of the current therapy for Pompe disease. Upper extremity MMT is related to flexion and extension of the shoulders and elbows, whereas lower extremity MMT is related to flexion and extension of the hips and knees.

### Selection of PROs

The Amicus PAB for Pompe disease was launched in 2008 and meets annually or biannually to address a predetermined agenda relevant to patient advocacy. PAB candidates are referred by patient organization leaders, disease community leaders, Amicus staff familiar with community members through various meetings and events, and other referral sources. The selection process includes candidates completing an application that captures their understanding and commitment to their disease community, in conjunction with at least one phone interview, followed by a review process. The recommended number of members is between 5 and 10 for a minimum 2-year commitment. Members are selected to reflect the diversity of those affected by Pompe disease to ensure representation of the entire population. Members provide insight about disease experience that may affect the development of clinical, patient advocacy, patient services, and reimbursement programs at Amicus. Meeting topics may include, but are not limited to, clinical trials, disease management, physician-patient communication, access to health care, educational materials or resource reviews, and public policy issues. In the past, Amicus PABs have informed the cross-functional disease program team and provided direction on a range of issues, including clinical trial and protocol design, disease awareness education and support initiatives, market research, and possible access to treatment, among other key activities. The primary authors, AEM and JG and SD, reviewed PROMIS questionnaires of relevance to the patient population with Pompe disease based on reported signs and symptoms relevant to this disease. From discussions with author PK, noted clinical expert and physician managing Pompe disease, final selection of chosen PROMIS instruments was made.

The Amicus PAB for Pompe disease meets annually or biannually to address a predetermined agenda. The selection process for advisors includes candidates completing an application and interview process that captures their understanding and commitment to their knowledge of Pompe disease and the Pompe disease community. Members provide insight about disease experience that may affect the development of clinical, patient advocacy, patient services, and reimbursement programs at Amicus. Meeting topics may include, but are not limited to, clinical trials, disease management, physician-patient communication, access to health care, educational materials or resource reviews, and public policy issues. In the past, Amicus PABs have informed.

the cross-functional disease program team and provided direction on a range of issues, including clinical trial and protocol design, disease awareness education and support initiatives, market research, and possible access to treatment, among other key activities.

At the April 2018 PAB meeting, members were given time to review each of the selected PROMIS questionnaires and were individually surveyed on a series of questions meant to measure if those questionnaires accurately represented a symptom and/or sign of an unmet need in Pompe disease. Members were asked to rate the overall relevance of the five PROMIS questionnaires to Pompe disease and the importance of each question in each questionnaire. Members were also asked whether any other unmet needs could have been addressed with the questionnaires and how the questionnaires could be improved for future studies. The Pain Interference SF 8a was not evaluated by the PAB.

### Statistical analysis

For the descriptive statistics, categorical variables were summarized by frequency and percentage for each response category (number of patients, %) and continuous variables were summarized using means, medians, minimum, maximum, and standard deviations.

Data were analyzed as observed. Missing data were not imputed.

If any of the assessments were collected more than once, the last record was used for descriptive statistics summary and further analysis. If any of the MMT values were missing or any questions in the PROMIS questionnaires were unanswered, those patients were excluded from the analysis.

Both Pearson correlation coefficient and Spearman correlation coefficient were calculated between physical outcome measures and PROMIS questionnaires. Two-sided statistical tests for correlations were performed at the 0.05 significance level. There was no adjustment for multiplicity.

## Results

Thirty patients (12 male,18 female) completed the PROMIS questionnaires during clinical visits to the Duke University Pompe Clinical and Research Program. The mean age of the patients was 51 years (range, 18–79 years). The mean age at the time of diagnosis of LOPD was 44 years, and the average duration from the time of diagnosis to the time of the study was 7 years. The average age at onset of signs and symptoms of muscle and respiratory disease manifestations was 31 (range, 1.25–52 years) and 43 (range, 19–62 years) years, respectively. The duration of enzyme replacement therapy was 1 to 12 years. All patients who participated in the study were ambulatory.

Table 3 shows the number of patients who completed each PROMIS questionnaire, the average raw score, the standard deviation from the mean, the median of the scores, and the minimum and maximum scores from each questionnaire (see Appendix 1 for the mean score for each question).

**Table 3:** Average Raw Scores for PROMIS Questionnaires

	Number of Patients	Mean	Standard Deviation	Median	Minimum	Maximum
Pain Interference	29	16.72	9.180	16.00	8	35
Fatigue	29	23.48	8.671	22.00	8	40
Upper Extremity	30	25.10	7.174	25.00	13	35
Physical Function	30	71.47	13.761	70.50	44	100
Dyspnea	30	24.96	19.099	22.80	0	67.6

*PROMIS* Patient-Reported Outcomes Measurement Information System

### 6-minute walk distance and upper extremity short form 7a

**Fig. 1:**
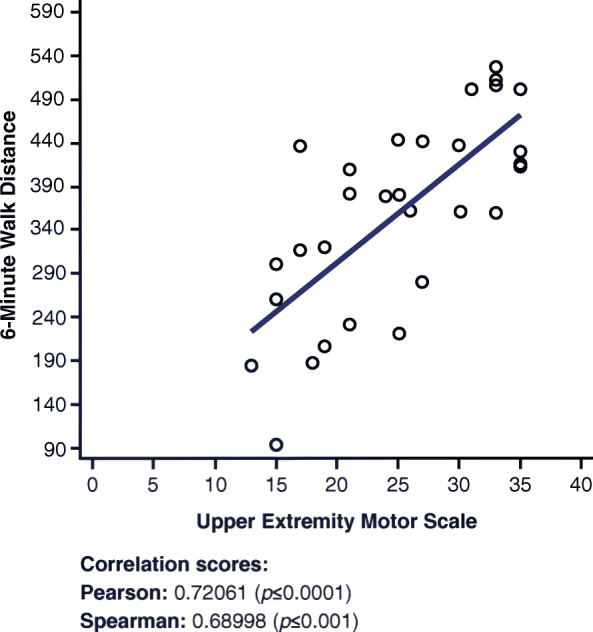
Correlations between 6-Minute Walk Distance and Upper Extremity Short Form 7a

A moderate to strong positive correlation is observed in Fig. 1 between the 6MWD and Upper Extremity SF 7a (r = 0.72061, *p* ≤ 0.0001). Similar trends in correlations of the % Predicted 6MWD and the Upper Extremity SF 7a (r = 0.58494, *p* = 0.0007) are observed. For more details regarding the correlations of % Predicted 6MWD and Upper Extremity SF 7a, see Appendix 2, All Correlation Scores.

### Overall manual muscle test and upper extremity short form 7a

**Fig. 2:**
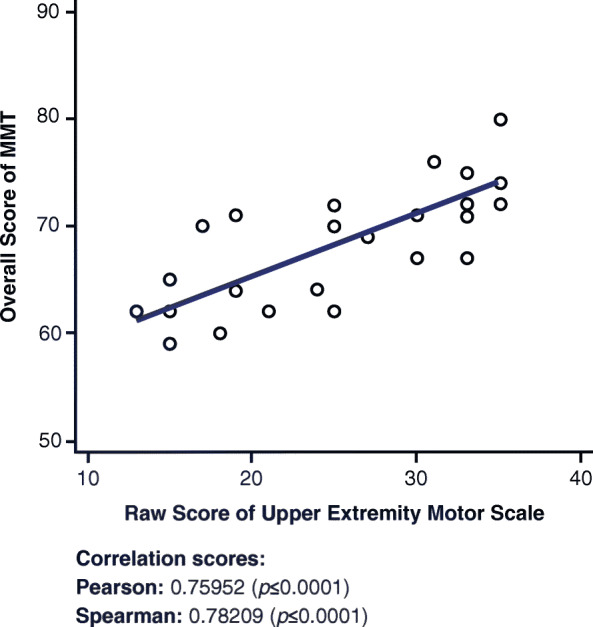
Correlations between Overall MMT and Upper Extremity Short Form 7a. MMT = Manual Muscle Test

A strong positive correlation is observed in Fig. 2 between the overall MMT score and the Upper Extremity SF 7a (r = 0.75952, *p* ≤ 0.0001).

### Upper (extremity) manual muscle test and upper extremity short form 7a

**Fig. 3:**
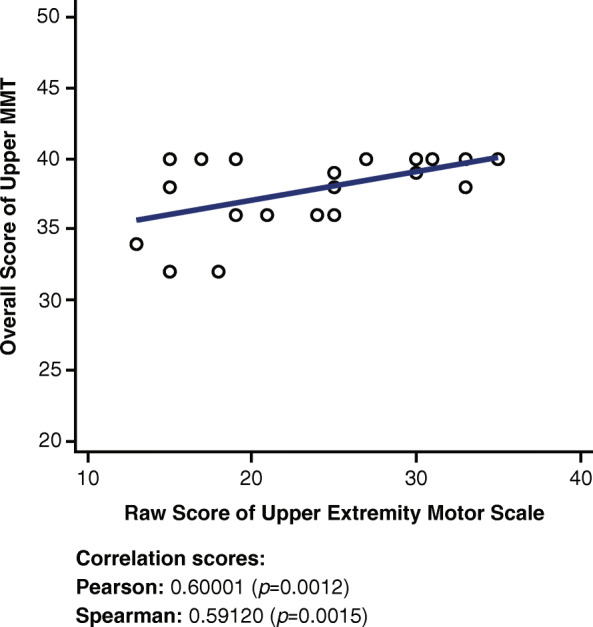
Correlations between Upper MMT and Upper Extremity Short Form 7a. MMT = Manual Muscle Test

A moderate positive correlation is observed in Fig. 3 between the upper MMT score and the Upper Extremity SF 7a (r = 0.60001, *p* = 0.0012).

### Lower (extremity) manual muscle test and upper extremity short form 7a

**Fig. 4:**
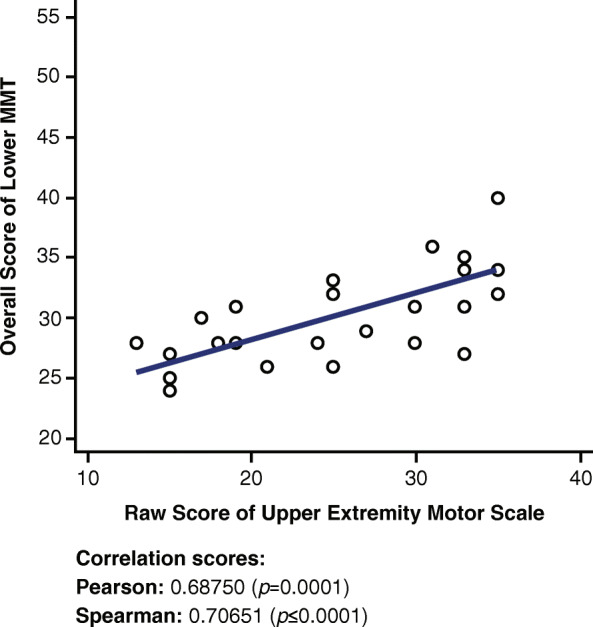
Correlations between Lower MMT and Upper Extremity Short Form 7a. MMT = Manual Muscle Test

A strong to moderate positive correlation is observed in Fig. 4 between the lower MMT score and the Upper Extremity SF 7a (r = 0.68750, *p* = 0.0001).

### Overall manual muscle test and physical function short form 20a

**Fig. 5:**
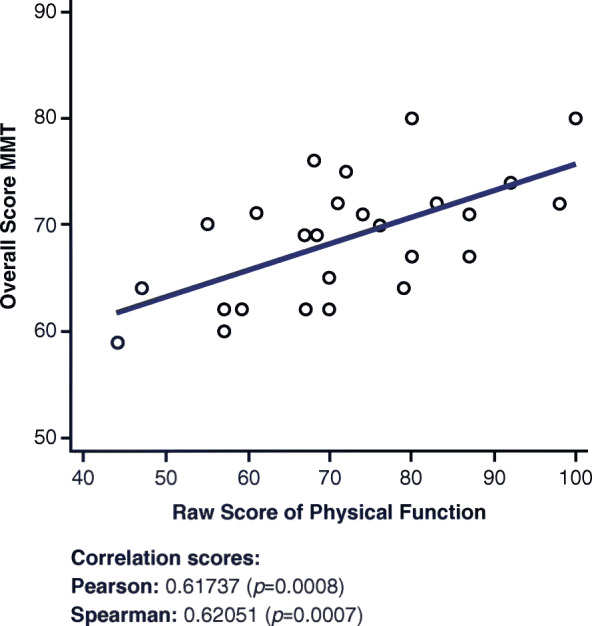
Correlations between Overall MMT and Physical Function Short Form 20a. MMT = Manual Muscle Test

A moderate positive correlation is observed in Fig. 5 between the overall MMT score and the Physical Function SF 20a questionnaire (r = 0.61737, *p* = 0.0008).

### Upper manual muscle test and physical function short form 20a

**Fig. 6:**
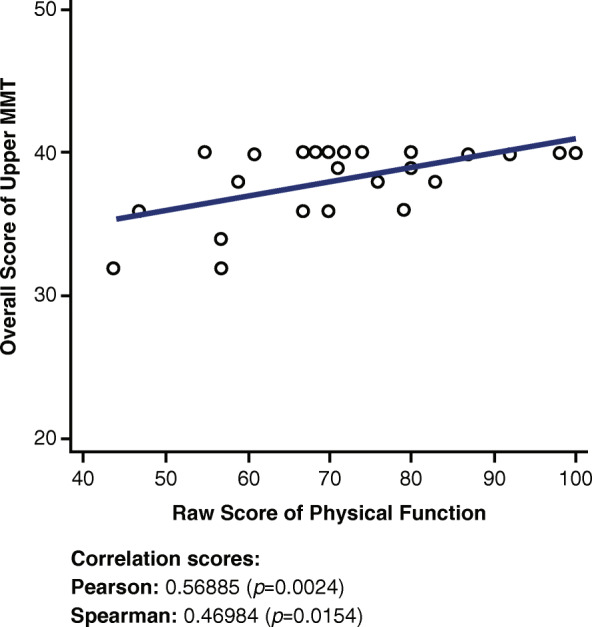
Correlations between Upper MMT and Physical Function Short Form 20a. MMT = Manual Muscle Test

A low to moderate positive correlation is observed in Fig. 6 between the upper MMT score and the Physical Function SF 20a questionnaire (r = 0.56885, *p* = 0.0024).

### Lower manual muscle test and physical function short form 20a

**Fig. 7:**
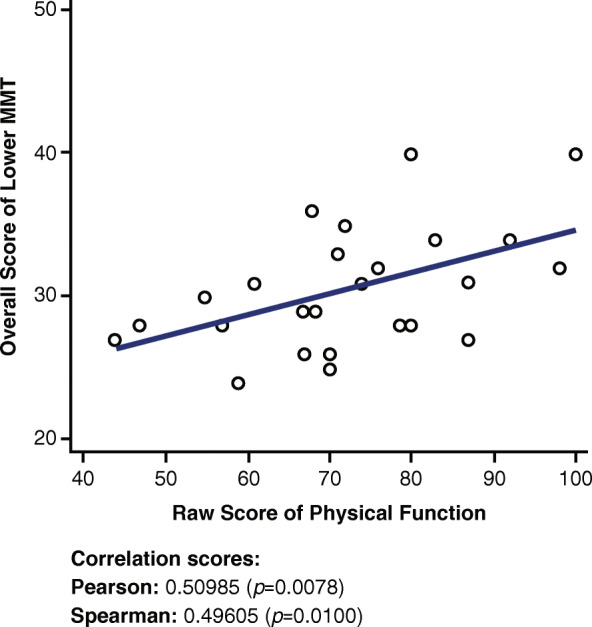
Correlations between Lower MMT and Physical Function Short Form 20a. MMT = Manual Muscle Test

A low to moderate positive correlation is observed in Fig. 7 between the lower MMT score and the Physical Function SF 20a questionnaire (r = 0.50985, *p* = 0.0078).

### Summary of all correlations

All Correlation Scores in Appendix 2 shows each of the clinical outcome assessments and each of the PROMIS questionnaires. It includes the Pearson and Spearman correlation coefficients as well as their respective *p* values. Figures 1, 2, 3, 4, 5, 6 and 7 demonstrate only moderate to strong correlations. Aside from the correlations shown in Figs. 1, 2, 3, 4, 5, 6 and 7, the correlations between 6MWD and Physical Function SF 20a were statistically significant. 6MWD and MMT showed no correlation with Pain Interference SF 8a, Fatigue SF 8a, or Dyspnea SF 10a, and the % Predicted FVC showed no correlation with any PROMIS questionnaires (Appendix 2). It cannot be determined what is the clinical meaningfulness of these data.

### Patient advisory board survey

The 6 April 2018 PAB meeting had eight members in attendance: three people living with LOPD, four people who are caregivers for people living with Pompe disease (adults and children), and one person who is a caregiver and a Patient Advocacy Organization leader.

**Fig. 8:**
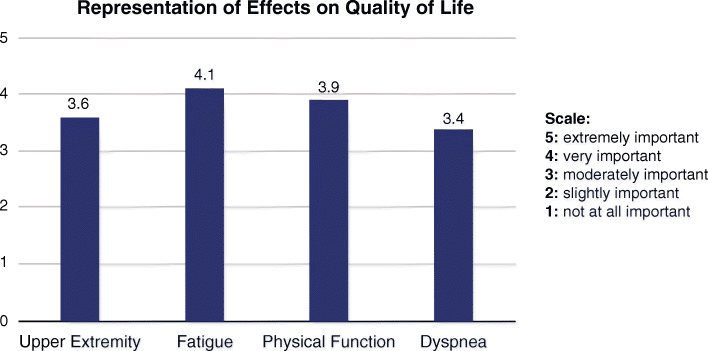
Patient Advisory Board discussion on the importance of PROMIS questions to health-related quality of life. PROMIS: Patient-Reported Outcomes Measurement Information System; SF: Short Form

PAB members were asked to rate the selected PROMIS scales on the importance in representing the effects on their QoL. Five of the eight PAB members (two patients, two caregivers, one caregiver and Patient Advocacy Organization leader) rated the importance of each domain (Upper Extremity, Fatigue, Physical Function, Dyspnea) (Fig. 8). Overall, the patient advisors rated the PROMIS scales as important to representing the effects on their HRQoL.

The same five PAB members also responded about whether the PROMIS questions accurately represented a symptom for which there is an unmet need in Pompe disease. Results of the survey are described in Table 4. The Pain Interference SF 8a questionnaire was not reviewed by the PAB, which is a limitation of the study (Additional file 2).

**Table 4:** PROMIS Questionnaire Versus Patient Advisory Board Responses

Scale	Summary of PAB Response
Upper Extremity SF 7a	7 of 7 questions rated addressing an unmet need
Fatigue SF 8a	8 of 8 questions rated addressing an unmet need
Physical Function SF 20a	19 of 20 questions rated addressing an unmet need
Dyspnea SF 10a	9 of 10 questions rated addressing an unmet need

*PAB* Patient Advisory Board, *PROMIS* Patient-Reported Outcomes Measurement Information System, *SF* Short Form

## Discussion

The data included in Results present moderate to strong correlations of five preselected PROMIS questionnaires with specific clinical outcome measures in a cross-sectional study of patients with LOPD.

The 6MWD is a clinical outcome assessment that measures the distance an individual can walk in 6 min. The relationship between the 6MWD and the Physical Function SF 20a is moderately correlated and statistically significant, as noted in Appendix 2. This relationship is clinically meaningful. The correlation between the 6MWD and the Upper Extremity SF 7a, as shown in Fig. 1, was expected because some of the questions in the patient questionnaire account for walking abilities. Questions such as “are you able to carry a heavy object” or “are you able to carry a shopping bag” assume that the patient is carrying the object somewhere and thus walking to that place. By accounting for the ability to walk, the Upper Extremity SF 7a questionnaire effectively correlates with the 6MWD. Based on the correlations between individual questions in the Upper Extremity SF 7a with the different clinical outcome measures, five of seven questions were positively correlated with the 6MWD. This means that the higher the score on the Upper Extremity SF 7a questionnaire, the more likely the patient can walk farther (measured in meters) in 6 min. As expected, similar trends in correlation of the % Predicted 6MWD and the Upper Extremity SF 7a (r = 0.58494, *p* = 0.0007) were observed, strengthening the meaningfulness of the data. For more details regarding the correlations of % Predicted 6MWD and the Upper Extremity SF 7a, see Appendix 2.

The MMT is a clinical outcome measure that accounts for the flexion, extension, and abduction of the shoulders, elbows, hips, and knees. The Upper Extremity SF 7a questionnaire measures the functionality of the same muscles by asking questions about the patient’s ability to carry objects or reach different body parts. Therefore, this correlation was expected (Fig. 2). This positive correlation suggests that the higher the score on the Upper Extremity SF 7a, the higher the functionality of the muscles, leading to a higher score on the overall MMT. Considering how the Upper Extremity SF 7a questionnaire is composed, accounting for both lower and upper body functions, it is not surprising that the correlation between overall MMT and Upper Extremity SF 7a (r = 0.75952, *p* = ≤ 0.0001) was higher than that of both the lower (r = 0.68750, *p* = 0.0001) and the upper (r = 0.60001, *p* = 0.0012) MMTs individually.

The upper MMT is focused on the flexion, extension, and abduction of only the shoulders and elbows. Therefore, a correlation between the upper MMT and the Upper Extremity SF 7a was expected (Fig. 3) because the muscles tested in the upper MMT are directly responsible for the actions outlined in the Upper Extremity SF 7a. A positive correlation between the upper MMT and the Upper Extremity SF 7a suggests that the higher an individual score on the Upper Extremity SF 7a, the more functionality exhibited by the muscles on the upper MMT test.

Surprisingly, the correlation between the lower MMT and the Upper Extremity SF 7a (Fig. 4) was actually stronger than that between the upper MMT and the Upper Extremity SF 7a. This stronger correlation could be due to the questions mentioned previously regarding the ability to carry an object, which encompass the abilities of the lower body. Based on the individual questions that were correlated against all the clinical outcome measures (Appendix 3, Individual Correlations), numerous questions were correlated with the lower MMT as well as the upper MMT, which can help explain the higher overall correlation between the lower MMT and the Upper Extremity SF 7a. All seven questions in the Upper Extremity SF 7a questionnaire correlated with the lower MMT clinical measures, with five out of seven questions correlating more with the lower MMT than the upper MMT. This positive correlation suggests that a higher score on the Upper Extremity SF 7a questionnaire indicates that a patient is likely to have a higher score on the lower MMT test. These data are considered preliminary.

The correlation between the overall MMT and the Physical Function SF 20a (Fig. 5) was expected because of the similarity between assessments in the MMT and the Physical Function SF 20a scoring. The questions test similar capabilities, and some questions, such as the ability to “wash one’s back,” overlap between the two measures. The overall MMT has a higher correlation with the Physical Function SF 20a than the upper MMT and the lower MMT against the Physical Function SF 20a, as expected, because it accounts for both the upper and lower muscles, which together account for the ability to perform the tasks encountered in the physical function assessment.

The upper MMT has a stronger correlation with the Physical Function SF 20a than the lower MMT because of the questions in the Physical Function SF 20a assessment. Most of the questions on the Physical Function SF 20a questionnaire are geared toward the upper body because the lower muscles are not as engaged in “squeezing a new tube of toothpaste” or “being able to shampoo your hair.” The moderate positive correlation (Fig. 6) suggests that the more able-bodied a patient is, ranking higher on the Physical Function SF 20a scale, the higher the individual scores on upper MMT, marking a higher functionality of the upper body muscles.

The lower MMT has a lower correlation with the Physical Function SF 20a (Fig. 7) than the upper MMT because only five of the 14 questions in the Physical Function SF 20a questionnaire account for primarily lower body functions. These questions include activities such as vacuuming, yard work, getting in and out of a car, running a short distance, being able to get up from a toilet, and being able to transfer from a bed to a chair and back. Because most of the questions (nine of the 14) account for upper body work, it is expected that the correlation with the lower MMT will be less than that with the upper MMT.

The moderate to strong correlations across several important PRO measures with the MMT indicate that changes in the MMT are occurring with changes in important PRO measures of how patients feel and function, lending credibility to the fact that the MMT is reasonably likely to predict clinical benefit but not confirmed with the results in this study. The evidence provided in this report will not support the MMT as a validated surrogate endpoint for traditional regulatory approval, but it may provide additional evidence to propose the MMT as a surrogate marker reasonably likely to predict clinical benefit. In patient care environments that are under-resourced, PROs may be considered to provide other insight into muscle function.

Although the Fatigue SF 8a and the Dyspnea SF 10a did not show a correlation with the 6MWD (r = − 0.01041 and r = − 0.3487, respectively; Appendix 2), both the Physical Function SF 20a and the Upper Extremity SF 7a demonstrated a moderate to strong positive correlation with the 6MWD (r = 0.50602 and r = 0.72061, respectively). Additionally, although the Fatigue SF 8a and the Dyspnea SF 10a did not show a strong or moderate correlation with the overall MMT (r = − 0.27537 and r = − 0.13391, respectively), both the Physical Function SF 20a and the Upper Extremity SF 7a demonstrated a moderate to strong positive correlation with the overall MMT (r = 0.61737 and r = 0.75952, respectively).

Overall, these data support the construct and content validity of the PROMIS tools because they are supportive of the motor signs and symptoms of functional disability observed in patients with Pompe disease. In addition, the relationships manifested between certain clinical outcome assessments indicative of muscle weakness may be relevant to help support the functional relevance of these data in terms of impact on patient care. Prospective study and analysis of these PROs and others are encouraged for further study in a larger cohort of patients with Pompe disease.

Five of the eight patients who participated in the PAB were able to complete the PROMIS questionnaire survey and to rank the importance of four PROMIS questionnaires used in the study (Fig. 8). Altogether, the high scores for each of the PROMIS domains indicate that these questionnaires are reflective of the patient experience. Additionally, the patient advisors said the majority of the PROMIS scale questions did address an unmet need, suggesting that the PROMIS questionnaires demonstrate clinical relevance in Pompe disease.

The study has limitations. The PROMIS scales selected for analysis by the PAB were chosen by Amicus as measures clinically relevant to patients living with Pompe disease. The Pain Interference SF 8a was not correlated with any of the clinical outcome measures; therefore, the Pain Interference SF 8a questionnaire was not reviewed by the PAB, a clear limitation of the study. In addition, failure to include comparative PROs studied in patients with Pompe disease is a limitation of this study. The lower extremity should also have been assessed as a PROMIS short form in addition to the Upper Extremity SF 7a. Regardless, this study remains important because it demonstrates the construct and content validity of the PROMIS questionnaires that were used in Pompe disease.

## Conclusions

The Duke University Pompe Disease Clinical Research Program study gathered data on select PROMIS questionnaires and assessed correlations between PROs and clinical outcome assessments to understand the meaningfulness of the PRO to patients reporting signs and symptoms of Pompe disease. The correlations indicated that the PROMIS tools and the other clinical outcome assessments are moderately to strongly related, indicating that the clinical outcome assessments measure important concepts related to patient-reported experiences.

The PAB findings indicated that the PROMIS questionnaires are meaningful and address concepts important to PAB patients. The PAB survey determined the construct and content validity of the PROMIS questionnaire in the context of the tested clinical outcome assessments. The results of the PAB survey indicated that the patient advisors rated the PROMIS scales as important to representing the impact on their HRQoL and that most PROMIS scale questions did address an unmet need.

Supportive evidence of the clinical meaningfulness of PROMIS in the context of Pompe disease would allow for the use of PROMIS in larger studies and in future clinical trials as an appropriate clinical outcome assessment. The results from this study could be useful for diagnostic and prognostic purposes or for trial eligibility. Using these PROs provides further insight into understanding patient related disease specific signs and symptoms of patients with Pompe disease. Rather than enrichment, these PROs can be used to provide further insight into understanding patient related disease specific signs and symptoms. The PROs can then help inform future clinical trial designs accordingly. These will enrich protocol design by accurately representing changes in Pompe disease signs and symptoms over time. Potential advantages include with continued use in clinical trials could reduce the burden of longer clinical assessments tests / long term studies. Additionally, the results from the PAB can offer further support as to the clinical meaningfulness of the PROMIS questionnaires in LOPD.

## Supplementary information

**Additional file 1: Appendix 1.** Average scores for all promis questionnaire questions. **Appendix 2.** All correlation scores. **Appendix 3.** Individual correlations.

**Additional file 2.**

## Data Availability

All data supporting the findings of this study are available in the article and its supplemental materials.

## Abbreviations

6MWD: 6-Minute Walk Distance
ADL: Activities of daily living
FDA: US Food and Drug Administration
FVC: Forced vital capacity
GAA: Acid α-glucosidase
HRQoL: Health-related quality of life
IOPD: Infantile-onset Pompe disease
IRB: Institutional Review Board
LOPD: Late-onset Pompe disease
MMT: Manual Muscle Test
PAB: Patient Advisory Board
PRO: Patient-reported outcome
PROMIS®: Patient-Reported Outcomes Measurement Information System
SF: Short Form
