# Supplementary material for: Use of the patient-reported outcomes measurement information system (PROMIS®) to assess late-onset Pompe disease severity
Source: J Patient Rep Outcomes. 2020 Oct 9;4:83. doi: 10.1186/s41687-020-00245-2 (PMC7547055; doi:10.1186/s41687-020-00245-2)
Supplement: Supplementary file 1 — Additional file 1: Appendix 1. Average scores for all promis questionnaire questions. Appendix 2. All correlation scores. Appendix 3. Individual correlations. [file 41687_2020_245_MOESM1_ESM.docx]

| Table 1 | | | | | | | |
| --- | --- | --- | --- | --- | --- | --- | --- |
| Average Score for Pain Interference Short Form 8a | | | | | | | |
| (Patients with late-onset Pompe disease) | | | | | | | |
| Question | N | Mean | Standard Deviation | Median | Min | Max |  |
|  | | | | | | |  |
| 1. How much did pain interfere with your day to day activities? | 29 | 2.17 | 1.256 | 2.00 | 1 | 5 |  |
|  | | | | | | |  |
| 2. How much did pain interfere with work around the house? | 29 | 2.14 | 1.187 | 2.00 | 1 | 4 |  |
|  | | | | | | |  |
| 3. How much did pain interfere with your ability to participate in social activities | 29 | 1.97 | 1.149 | 2.00 | 1 | 4 |  |
|  | | | | | | |  |
| 4. How much did pain interfere with your enjoyment of life? | 29 | 2.07 | 1.193 | 2.00 | 1 | 4 |  |
|  | | | | | | |  |
| 5. How much did pain interfere with the things you usually do for fun? | 29 | 2.14 | 1.156 | 2.00 | 1 | 4 |  |
|  | | | | | | |  |
| 6. How much did pain interfere with your enjoyment of social activities? | 29 | 2.00 | 1.195 | 1.00 | 1 | 4 |  |
|  | | | | | | |  |
| 7. How much did pain interfere with your household chores? | 29 | 2.24 | 1.327 | 2.00 | 1 | 5 |  |
|  | | | | | | |  |
| 8. How much did pain interfere with your family life? | 29 | 2.00 | 1.195 | 2.00 | 1 | 5 |  |
|  | | | | | | |  |
| Raw Score | 29 | 16.72 | 9.180 | 16.00 | 8 | 35 |  |
|  | | | | | | |  |
| T Score | 29 | 52.33 | 10.678 | 55.80 | 40.7 | 69.2 |  |

# Appendix 1. Average Scores for all PROMIS Questionnaire Questions

|  |
| --- |
| 1=Not at all |
| 2=A little bit |
| 3=Somewhat |
| 4=Quite a bit |
| 5=Very much |

| Table 2. | | | | | | | |
| --- | --- | --- | --- | --- | --- | --- | --- |
| Average Score for Upper Extremity Short Form 7a | | | | | | | |
| (Patients with late-onset Pompe disease) | | | | | | | |
| Question | N | Mean | Standard Deviation | Median | Min | Max |  |
| 1. Are you able to carry a heavy object (over 10 pounds/5kg)? | 30 | 3.13 | 1.306 | 3.00 | 1 | 5 |  |
|  | | | | | | |  |
| 2. Are you able to wash your back? | 30 | 4.07 | 1.172 | 5.00 | 1 | 5 |  |
|  | | | | | | |  |
| 3. Are you able to put on and take off a coat or jacket? | 30 | 4.40 | 1.003 | 5.00 | 1 | 5 |  |
|  | | | | | | |  |
| 4. Are you able to carry a shopping bag or briefcase? | 30 | 4.23 | 0.858 | 4.00 | 2 | 5 |  |
|  | | | | | | |  |
| 5. Are you able to lift 10 pounds (5 kg) above your shoulder? | 30 | 3.10 | 1.539 | 3.00 | 1 | 5 |  |
|  | | | | | | |  |
| 6. Are you able to change a light bulb overhead? | 30 | 3.40 | 1.567 | 4.00 | 1 | 5 |  |
|  | | | | | | |  |
| 7. Are you able to pass a 20-pound (10 kg) turkey or ham to other people at the table? | 30 | 2.77 | 1.406 | 3.00 | 1 | 5 |  |
|  | | | | | | |  |
| Raw Score | 30 | 25.10 | 7.174 | 25.00 | 13 | 35 |  |
|  | | | | | | |  |
| T Score | 30 | 39.25 | 9.847 | 36.95 | 24.5 | 58.2 |  |
|  | | | | | | |  |

|  | |  |
| --- | --- | --- |
| 1=Unable to do | |  |
| 2=With much difficulty | |  |
| 3=With some difficulty | |  |
| 4=With a little difficulty | |  |
| 5=Without any difficulty | |  |
|  | |  |
|  | |  |
|  |  | |
|  |  | |

| Table 3 |
| --- |
| Average Score for Fatigue |
| (Patients with late-onset Pompe disease) |

| Question | N | Mean | Standard Deviation | Median | Min | Max |
| --- | --- | --- | --- | --- | --- | --- |
| 1. I feel fatigued | 27 | 3.33 | 1.074 | 3.00 | 2 | 5 |
|  | | | | | | |
| 2. I have trouble starting things because I am tired | 24 | 3.04 | 1.083 | 3.00 | 2 | 5 |
|  | | | | | | |
| 3. How run-down did you feel on average? | 26 | 2.96 | 1.113 | 3.00 | 2 | 5 |
|  | | | | | | |
| 4. How fatigued were you on average? | 26 | 2.96 | 1.038 | 3.00 | 2 | 5 |
|  | | | | | | |
| 5. How much were you bothered by your fatigue on average? | 25 | 3.20 | 1.080 | 3.00 | 2 | 5 |
|  | | | | | | |
| 6. To what degree did your fatigue interfere with your physical functioning? | 25 | 3.24 | 1.091 | 3.00 | 2 | 5 |
|  | | | | | | |
| 7. How often did you have to push yourself to get things done because of your fatigue? | 28 | 3.36 | 0.911 | 3.00 | 2 | 5 |
|  | | | | | | |
| 8. How often did you have trouble finishing things because of your fatigue? | 26 | 3.23 | 0.863 | 3.00 | 2 | 5 |
|  | | | | | | |
| Raw Score | 29 | 23.48 | 8.671 | 22.00 | 8 | 40 |
|  | | | | | | |
| T Score | 29 | 56.69 | 9.922 | 55.60 | 33.1 | 77.8 |
|  | | | | | | |

|  |
| --- |
| 1=Not at all |
| 2=A little bit |
| 3=Somewhat |
| 4=Quite a bit |
| 5=Very much |
|  |

| Table 4 | | | | | | | |
| --- | --- | --- | --- | --- | --- | --- | --- |
| Average Score for Physical Function Short Form 20a | | | | | | | |
| (Patients with late-onset Pompe disease) | | | | | | | |
| Question | N | Mean | Standard Deviation | Median | Min | Max |  |
|  | | | | | | |  |
| 1. Are you able to do chores such as vacuuming or yard work? | 30 | 3.27 | 1.143 | 3.00 | 1 | 5 |  |
|  | | | | | | |  |
| 2. Are you able to push open a heavy door? | 30 | 3.53 | 1.042 | 3.00 | 2 | 5 |  |
|  | | | | | | |  |
| 3. Are you able to dress yourself, including tying shoelaces and buttoning your clothes? | 29 | 4.48 | 0.829 | 5.00 | 2 | 5 |  |
|  | | | | | | |  |
| 4. Are you able to wash your back? | 30 | 4.03 | 1.245 | 4.50 | 1 | 5 |  |
|  | | | | | | |  |
| 5. Are you able to dry your back with a towel? | 30 | 4.30 | 1.055 | 5.00 | 1 | 5 |  |
|  | | | | | | |  |
| 6. Are you able to sit on the edge of a bed? | 30 | 4.83 | 0.379 | 5.00 | 4 | 5 |  |
|  | | | | | | |  |
| 7. Are you able to wash and dry your body? | 30 | 4.50 | 0.731 | 5.00 | 3 | 5 |  |
|  | | | | | | |  |
| 8. Are you able to get in and out of a car? | 30 | 3.97 | 1.066 | 4.00 | 1 | 5 |  |
|  | | | | | | |  |
| 9. Are you able to squeeze a new tube of toothpaste? | 30 | 4.93 | 0.365 | 5.00 | 3 | 5 |  |
|  | | | | | | |  |
| 10. Are you able to hold a plate full of food? | 30 | 4.77 | 0.430 | 5.00 | 4 | 5 |  |
|  | | | | | | |  |
| 11. Are you able to run a short distance, such as to catch a bus? | 30 | 1.90 | 1.373 | 1.00 | 1 | 5 |  |
|  | | | | | | |  |
| 12. Are you able to shampoo your hair? | 30 | 4.63 | 0.669 | 5.00 | 3 | 5 |  |
|  | | | | | | |  |
| 13. Are you able to sit on and get up from the toilet? | 29 | 4.14 | 0.915 | 4.00 | 2 | 5 |  |
|  | | | | | | |  |
| 14. Are you able to transfer from a bed to a chair and back? | 29 | 4.24 | 1.023 | 5.00 | 1 | 5 |  |
|  | | | | | | |  |
| 15. Does your health now limit you in doing vigorous activities, such as running, lifting heavy objects, participating in strenuous sports? | 30 | 1.47 | 1.074 | 1.00 | 1 | 5 |  |
|  | | | | | | |  |
| 16. Does your health now limit you in bending, kneeling, or stooping? | 30 | 2.40 | 1.133 | 2.00 | 1 | 5 |  |
|  | | | | | | |  |
| 17. Does your health limit you in lifting or carrying groceries? | 29 | 3.03 | 1.017 | 3.00 | 2 | 5 |  |
|  | | | | | | |  |
| 18. Does your health now limit you in doing two hours of physical labor? | 30 | 2.00 | 1.083 | 2.00 | 1 | 5 |  |
|  | | | | | | |  |
| 19. Does your health now limit you in walking more than a mile (1.6 km)? | 30 | 2.33 | 1.322 | 2.00 | 1 | 5 |  |
|  | | | | | | |  |
| 20. Does your health now limit you in climbing one flight of stairs? | 30 | 2.83 | 1.147 | 3.00 | 1 | 5 |  |
|  | | | | | | |  |
| Raw Score | 30 | 71.47 | 13.761 | 70.50 | 44 | 100 |  |
|  | | | | | | |  |
| T Score | 30 | 38.18 | 7.891 | 37.00 | 26.2 | 62.7 |  |

|  |
| --- |
| 1=Unable to do |
| 2=With much difficulty |
| 3=With some difficulty |
| 4=With a little difficulty |
| 5=Without any difficulty |
|  |
|  |

| Table 5 | | | | | | | |
| --- | --- | --- | --- | --- | --- | --- | --- |
| Average Score for Dyspnea Severity | | | | | | | |
| (Patients with late-onset Pompe disease) | | | | | | | |
| Question | N | Mean | Standard Deviation | Median | Min | Max |  |
| 1. Dressing yourself without help | 30 | 0.47 | 0.629 | 0.00 | 0 | 2 |  |
|  | | | | | | |  |
| 2. Walking 50 steps/paces on flat ground at a normal speed without stopping | 29 | 0.79 | 0.774 | 1.00 | 0 | 2 |  |
|  | | | | | | |  |
| 3. Walking up 20 stairs (2 flights) without stopping | 21 | 1.90 | 0.944 | 2.00 | 0 | 3 |  |
|  | | | | | | |  |
| 4. Preparing meals | 28 | 0.21 | 0.499 | 0.00 | 0 | 2 |  |
|  | | | | | | |  |
| 5. Washing dishes | 29 | 0.21 | 0.559 | 0.00 | 0 | 2 |  |
|  | | | | | | |  |
| 6. Sweeping or mopping | 23 | 0.61 | 0.783 | 0.00 | 0 | 3 |  |
|  | | | | | | |  |
| 7. Making a bed | 25 | 0.52 | 0.653 | 0.00 | 0 | 2 |  |
|  | | | | | | |  |
| 8. Lifting something weighing 10-20 lbs (about 4.5-9kg, like a large bag of groceries) | 23 | 1.04 | 0.976 | 1.00 | 0 | 3 |  |
|  | | | | | | |  |
| 9. Carrying something weighing 10-20 lbs (about 4.5-9kg, like a large bag of groceries) from one room to another | 22 | 1.36 | 0.953 | 1.00 | 0 | 3 |  |
|  | | | | | | |  |
| 10. Walking (faster than your usual speed) for 1/2 mile (almost 1 km) without stopping | 19 | 1.79 | 0.918 | 2.00 | 0 | 3 |  |
|  | | | | | | |  |
| 11. Taking a bath without help | 22 | 0.32 | 0.646 | 0.00 | 0 | 2 |  |
|  | | | | | | |  |
| 12. Taking a shower | 30 | 0.33 | 0.606 | 0.00 | 0 | 2 |  |
|  | | | | | | |  |
| 13. Putting on socks or stockings | 30 | 0.43 | 0.679 | 0.00 | 0 | 2 |  |
|  | | | | | | |  |
| 14. Standing for at least 5 minutes | 30 | 0.40 | 0.814 | 0.00 | 0 | 3 |  |
|  | | | | | | |  |
| 15. Walking 10 steps/paces on flat ground at a normal speed without stopping | 29 | 0.34 | 0.670 | 0.00 | 0 | 2 |  |
|  | | | | | | |  |
| 16. Walking 1/2 mile (almost 1 km) on flat ground at a normal speed without stopping | 20 | 1.15 | 0.933 | 1.00 | 0 | 3 |  |
|  | | | | | | |  |
| 17. Walking up 5 stairs without stopping | 27 | 0.96 | 1.091 | 1.00 | 0 | 3 |  |
|  | | | | | | |  |
| 18. Walking up 10 stairs (1 flight) without stopping | 22 | 1.59 | 1.008 | 1.50 | 0 | 3 |  |
|  | | | | | | |  |
| 19. Walking up 30 stairs (3 flights) without stopping | 14 | 2.00 | 0.961 | 2.00 | 0 | 3 |  |
|  | | | | | | |  |
| 20. Lifting something weighing less than 5 lbs (about 2 kg, like a houseplant) | 30 | 0.33 | 0.547 | 0.00 | 0 | 2 |  |
|  | | | | | | |  |
| 21. Lifting something weighing 5-10 lbs (about 2-4.5 kg, like a basket of clothes) | 28 | 0.68 | 0.863 | 0.00 | 0 | 3 |  |
|  | | | | | | |  |
| 22. Lifting something weighing more than 20 lbs (about 9 kg, like a medium-sized suitcase) | 20 | 1.35 | 1.137 | 1.00 | 0 | 3 |  |
|  | | | | | | |  |
| 23. Carrying something weighing less than 5 lbs (about 2 kg, like a houseplant) from one room to another | 29 | 0.41 | 0.628 | 0.00 | 0 | 2 |  |
|  | | | | | | |  |
| 24. Carrying something weighing 5-10 lbs (about 2-4.5 kg, like a basket of clothes) from one room to another | 26 | 0.92 | 0.891 | 1.00 | 0 | 3 |  |
|  | | | | | | |  |
| 25. Getting in or out of a car | 29 | 0.38 | 0.561 | 0.00 | 0 | 2 |  |
|  | | | | | | |  |
| 26. Dining out | 29 | 0.21 | 0.491 | 0.00 | 0 | 2 |  |
|  | | | | | | |  |
| 27. Low-intensity leisure activity (gardening, etc.) | 23 | 0.74 | 0.752 | 1.00 | 0 | 2 |  |
|  | | | | | | |  |
| 28. Moderate-intensity leisure activity (bicycling on level terrain, etc.) | 18 | 1.39 | 0.979 | 1.00 | 0 | 3 |  |
|  | | | | | | |  |
| 29. Walking (faster than your usual speed) for 50 steps without stopping | 20 | 1.45 | 1.050 | 1.00 | 0 | 3 |  |
|  | | | | | | |  |
| 30. Walking (faster than your usual speed) for at least 1 mile (a little more than 1.5 km) without stopping | 14 | 1.79 | 1.051 | 2.00 | 0 | 3 |  |
|  | | | | | | |  |
| 31. Singing or humming | 26 | 0.54 | 0.582 | 0.50 | 0 | 2 |  |
|  | | | | | | |  |
| 32. Talking while walking | 28 | 1.32 | 0.723 | 1.00 | 0 | 2 |  |
|  | | | | | | |  |
| 33. Scrubbing the floor or counter | 25 | 0.64 | 0.810 | 0.00 | 0 | 3 |  |
|  | | | | | | |  |
| Raw Score | 30 | 24.96 | 19.099 | 22.80 | 0 | 67.6 |  |
|  | | | | | | |  |
| T Score | 30 | 39.70 | 10.285 | 38.60 | 24.1 | 64.2 |  |
|  | | | | | | |  |

|  |
| --- |
| 0=No shortness of breath |
| 1=Mildly short of breath |
| 2=Moderately short of breath |
| 3=Severely short of breath |
| 99=I did not do this in the past 7 days |
|  |

# Appendix 2. All Correlation Scores

| Correlation Evaluated | Pearson Values | Spearman Values |
| --- | --- | --- |
| 6MWD versus Pain Interference SF 8a | -0.10392, *p* = 0.5916 | -0.05855, p = 0.7629 |
| % Predicted 6MWD versus Pain Interference SF 8a | -0.23324, *p* = 0.2233 | -0.18869, *p* = 0.3270 |
| Overall MMT versus Pain Interference SF 8a | -0.27752, *p* = 0.1792 | -0.26400, *p* = 0.2022 |
| Upper MMT versus Pain Interference SF 8a | -0.26703, *p* = 0.1969 | -0.18245, *p* = 0.3827 |
| Lower MMT versus Pain Interference SF 8a | -0.22736, *p* = 0.2744 | -0.18695, *p* = 0.3709 |
| % Predicted FVC [Upright] versus Pain Interference SF 8a | -0.27422, *p* = 0.1500 | -0.16906, *p* = 0.3806 |
| % Predicted FVC [Supine] versus Pain Interference SF 8a | -0.05145, *p* = 0.8071 | 0.03781, *p* = 0.8576 |
| 6MWD versus Upper Extremity SF 7a | 0.72061, *p* ≤ 0.0001 | 0.68998, *p* ≤ 0.0001 |
| % Predicted 6MWD versus Upper Extremity SF 7a | 0.58494, *p* = 0.0007 | 0.53206, *p* = 0.0025 |
| Overall MMT versus Upper Extremity SF 7a | 0.75952, *p* ≤ 0.0001 | 0.78209, *p* ≤ 0.0001 |
| Upper MMT versus Upper Extremity SF 7a | 0.60001, *p* = 0.0012 | 0.59120, *p* = 0.0015 |
| Lower MMT versus Upper Extremity SF 7a | 0.68750, *p* = 0.0001 | 0.70651, *p* ≤ 0.0001 |
| % Predicted FVC [Upright] versus Upper Extremity SF 7a | 0.19465, *p* = 0.3027 | 0.13105, *p* = 0.4900 |
| % Predicted FVC [Supine] versus Upper Extremity SF 7a | 0.20180, *p* = 0.3334 | 0.17775, *p* = 0.3953 |
| 6MWD versus Fatigue SF 8a | -0.01041, *p* = 0.9573 | -0.00468, *p* = 0.9808 |
| % Predicted 6MWD versus Fatigue SF 8a | -0.01213, *p* = 0.9502 | 0.04438, *p* = 0.8192 |
| Overall MMT versus Fatigue SF 8a | -0.27537, *p* = 0.1733 | -0.23772, *p* = 0.2422 |
| Upper MMT versus Fatigue SF 8a | -0.02225, *p* = 0.9141 | -0.10460, *p* = 0.6111 |
| Lower MMT versus Fatigue SF 8a | -0.36719, *p* = 0.0650 | -0.28788, *p* = 0.1538 |
| % Predicted FVC [Upright] versus Fatigue SF 8a | -0.15719, *p* = 0.4154 | -0.07437, *p* = 0.7014 |
| 6MWD versus Physical Function SF 20a | 0.50602, *p* = 0.0043 | 0.48274, *p* = 0.0069 |
| % Predicted 6MWD versus Physical Function SF 20a | 0.35612, *p* = 0.0534 | 0.31031, *p* = 0.0951 |
| Overall MMT versus Physical Function SF 20a | 0.61737, *p* = 0.0008 | 0.62051, *p* = 0.0007 |
| Upper MMT versus Physical Function SF 20a | 0.56885, *p* = 0.0024 | 0.46984, *p* = 0.0154 |
| Lower MMT versus Physical Function SF 20a | 0.50985, *p* = 0.0078 | 0.49605, *p* = 0.0100 |
| % Predicted FVC [Upright] versus Physical Function SF 20a | 0.22629, *p* = 0.2292 | 0.11565, *p* = 0.5428 |
| 6MWD versus Dyspnea SF 10a | -0.03487, *p* = 0.8549 | -0.06098, *p* = 0.7489 |
| % Predicted 6MWD versus Dyspnea SF 10a | -0.07554, *p* = 0.6916 | 0.03427, p = 0.8573 |
| Overall MMT versus Dyspnea SF 10a | -0.13391, *p* = 0.5143 | -0.12292, *p* = 0.5497 |
| Upper MMT versus Dyspnea SF 10a | -0.19884, *p* = 0.3302 | -0.17493, *p* = 0.3927 |
| Lower MMT versus Dyspnea SF 10a | -0.06502, *p* = 0.7523 | 0.00361, *p* = 0.9860 |
| % Predicted FVC [Upright] versus Dyspnea SF 10a | -0.39084, *p* = 0.0327 | -0.22340, *p* = 0.2354 |
| % Predicted FVC [Supine] versus Dyspnea SF 10a | -0.26206, *p* = 0.2057 | -0.22075, *p* = 0.2890 |

6MWD = 6-Minute Walk Distance; FVC = forced vital capacity; MMT = Manual Muscle Test; SF = Short Form

# Appendix 3. Individual Correlations

| Table 6 |
| --- |
| Correlation between Each Pain Interference Question Score vs. All the Parameters |
| (Patients with late-onset Pompe disease) |

|  | | | Pearson Correlation | | Spearman Correlation | |
| --- | --- | --- | --- | --- | --- | --- |
| Questions | Parameters | N | Coefficient | P-value | Coefficient | P-value |
|  | | | | | | |
| 1. How much did pain interfere with your day to day activities? | %Predicted FVC - upright | 29 | -0.3168 | 0.094 | -0.2029 | 0.291 |
|  | %Predicted FVC - supine | 25 | -0.0879 | 0.676 | -0.0082 | 0.969 |
|  | Total MMT | 26 | -0.2923 | 0.156 | -0.3146 | 0.126 |
|  | Upper MMT | 26 | -0.2624 | 0.205 | -0.1528 | 0.466 |
|  | Lower MMT | 26 | -0.2493 | 0.229 | -0.2491 | 0.230 |
|  | Six Minute Walk Distance | 29 | -0.1310 | 0.498 | -0.1471 | 0.446 |
|  | %Predicted 6MWD | 29 | -0.2439 | 0.202 | -0.2465 | 0.197 |
|  | | | | | | |
| 2. How much did pain interfere with work around the house? | %Predicted FVC - upright | 29 | -0.1801 | 0.350 | -0.0934 | 0.630 |
|  | %Predicted FVC - supine | 25 | -0.0307 | 0.884 | 0.0598 | 0.776 |
|  | Total MMT | 26 | -0.2768 | 0.180 | -0.2857 | 0.166 |
|  | Upper MMT | 26 | -0.2356 | 0.257 | -0.1447 | 0.490 |
|  | Lower MMT | 26 | -0.2430 | 0.242 | -0.2253 | 0.279 |
|  | Six Minute Walk Distance | 29 | -0.0325 | 0.867 | -0.0259 | 0.894 |
|  | %Predicted 6MWD | 29 | -0.1456 | 0.451 | -0.1370 | 0.479 |
|  | | | | | | |
| 3. How much did pain interfere with your ability to participate in social activities | %Predicted FVC - upright | 29 | -0.2186 | 0.255 | -0.1193 | 0.538 |
|  | %Predicted FVC - supine | 25 | 0.0099 | 0.962 | 0.1206 | 0.566 |
|  | Total MMT | 26 | -0.3371 | 0.099 | -0.3431 | 0.093 |
|  | Upper MMT | 26 | -0.4163 | 0.038* | -0.2385 | 0.251 |
|  | Lower MMT | 26 | -0.2278 | 0.273 | -0.2540 | 0.221 |
|  | Six Minute Walk Distance | 29 | -0.1760 | 0.361 | -0.1718 | 0.373 |
|  | %Predicted 6MWD | 29 | -0.3366 | 0.074 | -0.3111 | 0.100 |
|  | | | | | | |
| 4. How much did pain interfere with your enjoyment of life? | %Predicted FVC - upright | 29 | -0.2481 | 0.194 | -0.1569 | 0.416 |
|  | %Predicted FVC - supine | 25 | -0.1168 | 0.578 | -0.0433 | 0.837 |
|  | Total MMT | 26 | -0.2313 | 0.266 | -0.2659 | 0.199 |
|  | Upper MMT | 26 | -0.1832 | 0.381 | -0.0963 | 0.647 |
|  | Lower MMT | 26 | -0.2103 | 0.313 | -0.2453 | 0.237 |
|  | Six Minute Walk Distance | 29 | 0.0011 | 0.996 | 0.0129 | 0.947 |
|  | %Predicted 6MWD | 29 | -0.1047 | 0.589 | -0.0944 | 0.626 |
|  | | | | | | |
| 5. How much did pain interfere with the things you usually do for fun? | %Predicted FVC - upright | 29 | -0.2738 | 0.151 | -0.1918 | 0.319 |
|  | %Predicted FVC - supine | 25 | -0.0581 | 0.783 | 0.0102 | 0.961 |
|  | Total MMT | 26 | -0.2493 | 0.229 | -0.2632 | 0.204 |
|  | Upper MMT | 26 | -0.2709 | 0.190 | -0.2134 | 0.306 |
|  | Lower MMT | 26 | -0.1880 | 0.368 | -0.1885 | 0.367 |
|  | Six Minute Walk Distance | 29 | -0.1037 | 0.593 | -0.0719 | 0.711 |
|  | %Predicted 6MWD | 29 | -0.2360 | 0.218 | -0.2105 | 0.273 |
|  | | | | | | |
| 6. How much did pain interfere with your enjoyment of social activities? | %Predicted FVC - upright | 29 | -0.1860 | 0.334 | -0.0627 | 0.747 |
|  | %Predicted FVC - supine | 25 | 0.0267 | 0.899 | 0.1359 | 0.517 |
|  | Total MMT | 26 | -0.2651 | 0.200 | -0.2898 | 0.160 |
|  | Upper MMT | 26 | -0.2459 | 0.236 | -0.1370 | 0.514 |
|  | Lower MMT | 26 | -0.2220 | 0.286 | -0.2514 | 0.225 |
|  | Six Minute Walk Distance | 29 | -0.1287 | 0.506 | -0.0820 | 0.672 |
|  | %Predicted 6MWD | 29 | -0.2485 | 0.194 | -0.2068 | 0.282 |
|  | | | | | | |
| 7. How much did pain interfere with your household chores? | %Predicted FVC - upright | 29 | -0.3142 | 0.097 | -0.2001 | 0.298 |
|  | %Predicted FVC - supine | 25 | -0.0462 | 0.826 | 0.0387 | 0.854 |
|  | Total MMT | 26 | -0.2463 | 0.235 | -0.2672 | 0.197 |
|  | Upper MMT | 26 | -0.2038 | 0.328 | -0.1113 | 0.596 |
|  | Lower MMT | 26 | -0.2192 | 0.292 | -0.1941 | 0.353 |
|  | Six Minute Walk Distance | 29 | -0.1054 | 0.586 | -0.0784 | 0.686 |
|  | %Predicted 6MWD | 29 | -0.2037 | 0.289 | -0.1750 | 0.364 |
|  | | | | | | |
| 8. How much did pain interfere with your family life? | %Predicted FVC - upright | 29 | -0.3370 | 0.074 | -0.2298 | 0.231 |
|  | %Predicted FVC - supine | 25 | -0.0860 | 0.683 | -0.0180 | 0.932 |
|  | Total MMT | 26 | -0.2217 | 0.287 | -0.2561 | 0.217 |
|  | Upper MMT | 26 | -0.2291 | 0.271 | -0.1458 | 0.487 |
|  | Lower MMT | 26 | -0.1734 | 0.407 | -0.1986 | 0.341 |
|  | Six Minute Walk Distance | 29 | -0.1141 | 0.556 | -0.1328 | 0.492 |
|  | %Predicted 6MWD | 29 | -0.2595 | 0.174 | -0.2667 | 0.162 |

|  |
| --- |

6MWD = 6-Minute Walk Distance; FVC = forced vital capacity; MMT = Manual Muscle Test.

| Table 7 | | | | | | | |
| --- | --- | --- | --- | --- | --- | --- | --- |
| Correlation between Each Upper Extremity Question Score vs. All the Parameters | | | | | | | |
| (Patients with late-onset Pompe disease) | | | | | | | |
|  | | | | Pearson Correlation | | Spearman Correlation | |
| Questions | | Parameters | N | Coefficient | P-value | Coefficient | P-value |
|  | | | | | | | |
| 1. Are you able to carry a heavy object (over 10 pounds/5kg)? | | %Predicted FVC - upright | 30 | 0.0956 | 0.615 | 0.0302 | 0.874 |
|  | | %Predicted FVC - supine | 25 | 0.0500 | 0.812 | -0.0028 | 0.989 |
|  | | Total MMT | 26 | 0.7061 | 0.000* | 0.7291 | 0.000* |
|  | | Upper MMT | 26 | 0.5041 | 0.009* | 0.5166 | 0.007* |
|  | | Lower MMT | 26 | 0.6716 | 0.000* | 0.6885 | 0.000* |
|  | | Six Minute Walk Distance | 30 | 0.7309 | 0.000* | 0.6710 | 0.000* |
|  | | %Predicted 6MWD | 30 | 0.5299 | 0.003* | 0.5144 | 0.004* |
|  | | | | | | | |
| 2. Are you able to wash your back? | | %Predicted FVC - upright | 30 | 0.1904 | 0.314 | 0.1076 | 0.571 |
|  | | %Predicted FVC - supine | 25 | 0.2539 | 0.221 | 0.1796 | 0.390 |
|  | | Total MMT | 26 | 0.5364 | 0.005* | 0.5159 | 0.007* |
|  | | Upper MMT | 26 | 0.5566 | 0.003* | 0.4222 | 0.032* |
|  | | Lower MMT | 26 | 0.4054 | 0.040* | 0.4010 | 0.042* |
|  | | Six Minute Walk Distance | 30 | 0.2830 | 0.130 | 0.3418 | 0.064 |
|  | | %Predicted 6MWD | 30 | 0.2015 | 0.286 | 0.1917 | 0.310 |
|  | | | | | | | |
| 3. Are you able to put on and take off a coat or jacket? | | %Predicted FVC - upright | 30 | 0.2522 | 0.179 | 0.2063 | 0.274 |
|  | | %Predicted FVC - supine | 25 | 0.3348 | 0.102 | 0.2928 | 0.155 |
|  | | Total MMT | 26 | 0.5527 | 0.003* | 0.5973 | 0.001* |
|  | | Upper MMT | 26 | 0.3821 | 0.054 | 0.3573 | 0.073 |
|  | | Lower MMT | 26 | 0.5333 | 0.005* | 0.5446 | 0.004* |
|  | | Six Minute Walk Distance | 30 | 0.5315 | 0.003* | 0.4212 | 0.020* |
|  | | %Predicted 6MWD | 30 | 0.4145 | 0.023* | 0.2829 | 0.130 |
|  | | | | | | | |
| 4. Are you able to carry a shopping bag or briefcase? | | %Predicted FVC - upright | 30 | -0.0985 | 0.605 | -0.1471 | 0.438 |
|  | | %Predicted FVC - supine | 25 | -0.1663 | 0.427 | -0.1124 | 0.593 |
|  | | Total MMT | 26 | 0.5807 | 0.002* | 0.6300 | 0.001* |
|  | | Upper MMT | 26 | 0.5918 | 0.001* | 0.5955 | 0.001* |
|  | | Lower MMT | 26 | 0.4453 | 0.023* | 0.5252 | 0.006* |
|  | | Six Minute Walk Distance | 30 | 0.3151 | 0.090 | 0.3445 | 0.062 |
|  | | %Predicted 6MWD | 30 | 0.3442 | 0.063 | 0.3358 | 0.070 |
|  | | | | | | | |
| 5. Are you able to lift 10 pounds (5 kg) above your shoulder? | | %Predicted FVC - upright | 30 | 0.2465 | 0.189 | 0.1967 | 0.298 |
|  | | %Predicted FVC - supine | 25 | 0.2170 | 0.298 | 0.2018 | 0.333 |
|  | | Total MMT | 26 | 0.6533 | 0.000* | 0.6569 | 0.000* |
|  | | Upper MMT | 26 | 0.4884 | 0.011* | 0.4828 | 0.012* |
|  | | Lower MMT | 26 | 0.6081 | 0.001* | 0.6013 | 0.001* |
|  | | Six Minute Walk Distance | 30 | 0.7786 | 0.000* | 0.7618 | 0.000* |
|  | | %Predicted 6MWD | 30 | 0.6412 | 0.000* | 0.6173 | 0.000* |
|  | | | | | | | |
| 6. Are you able to change a light bulb overhead? | | %Predicted FVC - upright | 30 | 0.2792 | 0.135 | 0.2353 | 0.211 |
|  | | %Predicted FVC - supine | 25 | 0.2133 | 0.306 | 0.2534 | 0.222 |
|  | | Total MMT | 26 | 0.5214 | 0.006* | 0.5585 | 0.003* |
|  | | Upper MMT | 26 | 0.3849 | 0.052 | 0.3865 | 0.051 |
|  | | Lower MMT | 26 | 0.4883 | 0.011* | 0.4872 | 0.012* |
|  | | Six Minute Walk Distance | 30 | 0.6864 | 0.000* | 0.7115 | 0.000* |
|  | | %Predicted 6MWD | 30 | 0.5869 | 0.001* | 0.5340 | 0.002* |
|  | | | | | | | |
| 7. Are you able to pass a 20-pound (10 kg) turkey or ham to other people at the table? | | %Predicted FVC - upright | 30 | 0.0447 | 0.814 | -0.0511 | 0.789 |
|  | | %Predicted FVC - supine | 25 | 0.1503 | 0.473 | 0.1057 | 0.615 |
|  | | Total MMT | 26 | 0.7538 | 0.000* | 0.7555 | 0.000* |
|  | | Upper MMT | 26 | 0.5607 | 0.003* | 0.5879 | 0.002* |
|  | | Lower MMT | 26 | 0.7033 | 0.000* | 0.7129 | 0.000* |
|  | | Six Minute Walk Distance | 30 | 0.5729 | 0.001* | 0.5295 | 0.003* |
|  | | %Predicted 6MWD | 30 | 0.4625 | 0.010* | 0.4305 | 0.018* |
|  | |  |  |  |  |  |  |

|  |
| --- |

6MWD = 6-Minute Walk Distance; FVC = forced vital capacity; MMT = Manual Muscle Test.

| Table 8 |
| --- |
| Correlation between Each Fatigue Question Score vs. All the Parameters |
| (Patients with late-onset Pompe disease) |

|  | | | Pearson Correlation | | Spearman Correlation | |
| --- | --- | --- | --- | --- | --- | --- |
| Questions | Parameters | N | Coefficient | P-value | Coefficient | P-value |
|  | | | | | | |
| 1. I feel fatigued | %Predicted FVC - upright | 27 | -0.0883 | 0.661 | -0.0405 | 0.841 |
|  | %Predicted FVC - supine | 25 | -0.0371 | 0.870 | 0.0272 | 0.904 |
|  | Total MMT | 26 | -0.3231 | 0.124 | -0.3624 | 0.082 |
|  | Upper MMT | 26 | -0.0078 | 0.971 | -0.1156 | 0.591 |
|  | Lower MMT | 26 | -0.4578 | 0.024* | -0.4707 | 0.020* |
|  | Six Minute Walk Distance | 27 | -0.0039 | 0.985 | 0.0161 | 0.936 |
|  | %Predicted 6MWD | 27 | -0.0310 | 0.878 | -0.0465 | 0.818 |
|  | | | | | | |
| 2. I have trouble starting things because I am tired | %Predicted FVC - upright | 24 | -0.1806 | 0.399 | -0.0231 | 0.915 |
|  | %Predicted FVC - supine | 24 | -0.1281 | 0.601 | -0.0611 | 0.804 |
|  | Total MMT | 24 | -0.1540 | 0.505 | -0.2015 | 0.381 |
|  | Upper MMT | 24 | 0.0876 | 0.706 | 0.1178 | 0.611 |
|  | Lower MMT | 24 | -0.2628 | 0.250 | -0.3011 | 0.185 |
|  | Six Minute Walk Distance | 24 | -0.0463 | 0.830 | -0.0755 | 0.726 |
|  | %Predicted 6MWD | 24 | -0.0731 | 0.734 | -0.0654 | 0.761 |
|  | | | | | | |
| 3. How run-down did you feel on average? | %Predicted FVC - upright | 26 | -0.0492 | 0.811 | 0.0394 | 0.848 |
|  | %Predicted FVC - supine | 25 | 0.1039 | 0.654 | 0.1650 | 0.475 |
|  | Total MMT | 26 | 0.0517 | 0.815 | 0.0621 | 0.778 |
|  | Upper MMT | 26 | 0.1811 | 0.408 | 0.1430 | 0.515 |
|  | Lower MMT | 26 | -0.0636 | 0.773 | -0.0370 | 0.867 |
|  | Six Minute Walk Distance | 26 | 0.0415 | 0.840 | 0.0168 | 0.935 |
|  | %Predicted 6MWD | 26 | -0.0818 | 0.691 | -0.0660 | 0.749 |
|  | | | | | | |
| 4. How fatigued were you on average? | %Predicted FVC - upright | 26 | 0.0743 | 0.718 | 0.1234 | 0.548 |
|  | %Predicted FVC - supine | 25 | 0.1738 | 0.451 | 0.2463 | 0.282 |
|  | Total MMT | 26 | 0.0897 | 0.684 | 0.0709 | 0.748 |
|  | Upper MMT | 26 | 0.2676 | 0.217 | 0.2100 | 0.336 |
|  | Lower MMT | 26 | -0.0737 | 0.738 | -0.0521 | 0.813 |
|  | Six Minute Walk Distance | 26 | 0.1229 | 0.550 | 0.1105 | 0.591 |
|  | %Predicted 6MWD | 26 | 0.0040 | 0.985 | 0.0177 | 0.931 |
|  | | | | | | |
| 5. How much were you bothered by your fatigue on average? | %Predicted FVC - upright | 25 | 0.0868 | 0.680 | 0.1143 | 0.586 |
|  | %Predicted FVC - supine | 25 | 0.1075 | 0.652 | 0.1465 | 0.538 |
|  | Total MMT | 25 | 0.0952 | 0.673 | 0.0986 | 0.662 |
|  | Upper MMT | 25 | 0.3255 | 0.139 | 0.2863 | 0.197 |
|  | Lower MMT | 25 | -0.1092 | 0.629 | -0.0690 | 0.760 |
|  | Six Minute Walk Distance | 25 | 0.1472 | 0.483 | 0.1160 | 0.581 |
|  | %Predicted 6MWD | 25 | 0.0369 | 0.861 | 0.0400 | 0.849 |
|  | | | | | | |
| 6. To what degree did your fatigue interfere with your physical functioning? | %Predicted FVC - upright | 25 | -0.0342 | 0.871 | 0.0602 | 0.775 |
|  | %Predicted FVC - supine | 25 | 0.1569 | 0.509 | 0.2323 | 0.324 |
|  | Total MMT | 25 | 0.0812 | 0.719 | 0.0731 | 0.746 |
|  | Upper MMT | 25 | 0.2160 | 0.334 | 0.1549 | 0.491 |
|  | Lower MMT | 25 | -0.0451 | 0.842 | -0.0234 | 0.918 |
|  | Six Minute Walk Distance | 25 | -0.0159 | 0.940 | -0.0667 | 0.751 |
|  | %Predicted 6MWD | 25 | -0.1391 | 0.507 | -0.1423 | 0.498 |
|  | | | | | | |
| 7. How often did you have to push yourself to get things done because of your fatigue? | %Predicted FVC - upright | 28 | -0.1625 | 0.409 | -0.0871 | 0.659 |
|  | %Predicted FVC - supine | 25 | -0.0002 | 0.999 | 0.0490 | 0.824 |
|  | Total MMT | 26 | -0.1350 | 0.520 | -0.1322 | 0.529 |
|  | Upper MMT | 26 | -0.0205 | 0.923 | -0.0534 | 0.800 |
|  | Lower MMT | 26 | -0.1794 | 0.391 | -0.1330 | 0.526 |
|  | Six Minute Walk Distance | 28 | -0.0414 | 0.834 | -0.0340 | 0.863 |
|  | %Predicted 6MWD | 28 | -0.2036 | 0.299 | -0.1644 | 0.403 |
|  | | | | | | |
| 8. How often did you have trouble finishing things because of your fatigue? | %Predicted FVC - upright | 26 | -0.2087 | 0.306 | -0.1289 | 0.530 |
|  | %Predicted FVC - supine | 25 | -0.2020 | 0.380 | -0.0905 | 0.696 |
|  | Total MMT | 26 | -0.1386 | 0.528 | -0.1638 | 0.455 |
|  | Upper MMT | 26 | -0.0455 | 0.837 | -0.0680 | 0.758 |
|  | Lower MMT | 26 | -0.1700 | 0.438 | -0.1474 | 0.502 |
|  | Six Minute Walk Distance | 26 | -0.0619 | 0.764 | -0.0351 | 0.865 |
|  | %Predicted 6MWD | 26 | -0.1743 | 0.394 | -0.1398 | 0.496 |

|  |
| --- |
|  |

6MWD = 6-Minute Walk Distance; FVC = forced vital capacity; MMT = Manual Muscle Test.

| Table 9 | | | | | | | |
| --- | --- | --- | --- | --- | --- | --- | --- |
| Correlation between Each Physical Function Question Score vs. All the Parameters | | | | | | | |
| (Patients with late-onset Pompe disease) | | | | | | | |
|  | | | Pearson Correlation | | Spearman Correlation | |  |
| Questions | Parameters | N | Coefficient | P-value | Coefficient | P-value |  |
|  | | | | | | |  |
| 1. Are you able to do chores such as vacuuming or yard work? | %Predicted FVC - upright | 30 | 0.3009 | 0.106 | 0.1434 | 0.450 |  |
|  | %Predicted FVC - supine | 25 | 0.1198 | 0.569 | 0.0070 | 0.974 |  |
|  | Total MMT | 26 | 0.3841 | 0.053 | 0.4017 | 0.042* |  |
|  | Upper MMT | 26 | 0.2742 | 0.175 | 0.3236 | 0.107 |  |
|  | Lower MMT | 26 | 0.3653 | 0.066 | 0.2934 | 0.146 |  |
|  | Six Minute Walk Distance | 30 | 0.6024 | 0.000* | 0.5178 | 0.003* |  |
|  | %Predicted 6MWD | 30 | 0.4635 | 0.010* | 0.3866 | 0.035* |  |
|  | | | | | | |  |
| 2. Are you able to push open a heavy door? | %Predicted FVC - upright | 30 | 0.0148 | 0.938 | -0.0134 | 0.944 |  |
|  | %Predicted FVC - supine | 25 | 0.1053 | 0.616 | 0.0562 | 0.790 |  |
|  | Total MMT | 26 | 0.5765 | 0.002* | 0.5817 | 0.002* |  |
|  | Upper MMT | 26 | 0.4542 | 0.020* | 0.4670 | 0.016* |  |
|  | Lower MMT | 26 | 0.5226 | 0.006* | 0.5442 | 0.004* |  |
|  | Six Minute Walk Distance | 30 | 0.5517 | 0.002* | 0.5193 | 0.003* |  |
|  | %Predicted 6MWD | 30 | 0.4068 | 0.026* | 0.3886 | 0.034* |  |
|  | | | | | | |  |
| 3. Are you able to dress yourself, including tying shoelaces and buttoning your clothes? | %Predicted FVC - upright | 29 | 0.3361 | 0.075 | 0.2458 | 0.199 |  |
|  | %Predicted FVC - supine | 25 | 0.3535 | 0.090 | 0.2962 | 0.160 |  |
|  | Total MMT | 26 | 0.2431 | 0.242 | 0.1850 | 0.376 |  |
|  | Upper MMT | 26 | 0.3918 | 0.053 | 0.1347 | 0.521 |  |
|  | Lower MMT | 26 | 0.0940 | 0.655 | 0.0370 | 0.861 |  |
|  | Six Minute Walk Distance | 29 | 0.0368 | 0.850 | 0.0734 | 0.705 |  |
|  | %Predicted 6MWD | 29 | 0.0816 | 0.674 | -0.0289 | 0.882 |  |
|  | | | | | | |  |
| 4. Are you able to wash your back? | %Predicted FVC - upright | 30 | 0.0321 | 0.866 | -0.0336 | 0.860 |  |
|  | %Predicted FVC - supine | 25 | 0.2129 | 0.307 | 0.1166 | 0.579 |  |
|  | Total MMT | 26 | 0.5871 | 0.002* | 0.5736 | 0.002* |  |
|  | Upper MMT | 26 | 0.6671 | 0.000* | 0.4816 | 0.013* |  |
|  | Lower MMT | 26 | 0.4087 | 0.038* | 0.4438 | 0.023* |  |
|  | Six Minute Walk Distance | 30 | 0.2886 | 0.122 | 0.2919 | 0.118 |  |
|  | %Predicted 6MWD | 30 | 0.2162 | 0.251 | 0.1607 | 0.396 |  |
|  | | | | | | |  |
| 5. Are you able to dry your back with a towel? | %Predicted FVC - upright | 30 | 0.2111 | 0.263 | 0.1747 | 0.356 |  |
|  | %Predicted FVC - supine | 25 | 0.2570 | 0.215 | 0.1990 | 0.340 |  |
|  | Total MMT | 26 | 0.4406 | 0.024* | 0.4597 | 0.018* |  |
|  | Upper MMT | 26 | 0.5249 | 0.006* | 0.4148 | 0.035* |  |
|  | Lower MMT | 26 | 0.2920 | 0.148 | 0.2814 | 0.164 |  |
|  | Six Minute Walk Distance | 30 | 0.1771 | 0.349 | 0.2402 | 0.201 |  |
|  | %Predicted 6MWD | 30 | 0.1184 | 0.533 | 0.1133 | 0.551 |  |
|  | | | | | | |  |
| 6. Are you able to sit on the edge of a bed? | %Predicted FVC - upright | 30 | 0.0950 | 0.617 | 0.0052 | 0.978 |  |
|  | %Predicted FVC - supine | 25 | 0.0411 | 0.845 | 0.0303 | 0.886 |  |
|  | Total MMT | 26 | 0.3704 | 0.062 | 0.3781 | 0.057 |  |
|  | Upper MMT | 26 | 0.3827 | 0.054 | 0.3414 | 0.088 |  |
|  | Lower MMT | 26 | 0.2809 | 0.164 | 0.3143 | 0.118 |  |
|  | Six Minute Walk Distance | 30 | -0.0113 | 0.953 | 0.0052 | 0.978 |  |
|  | %Predicted 6MWD | 30 | -0.0670 | 0.725 | -0.1085 | 0.568 |  |
|  | | | | | | |  |
| 7. Are you able to wash and dry your body? | %Predicted FVC - upright | 30 | 0.1747 | 0.356 | 0.1270 | 0.504 |  |
|  | %Predicted FVC - supine | 25 | 0.1891 | 0.365 | 0.1773 | 0.396 |  |
|  | Total MMT | 26 | 0.1983 | 0.331 | 0.1914 | 0.349 |  |
|  | Upper MMT | 26 | 0.2854 | 0.158 | 0.1276 | 0.535 |  |
|  | Lower MMT | 26 | 0.1018 | 0.621 | 0.0266 | 0.898 |  |
|  | Six Minute Walk Distance | 30 | 0.0003 | 0.999 | 0.0531 | 0.781 |  |
|  | %Predicted 6MWD | 30 | -0.0554 | 0.771 | -0.0453 | 0.812 |  |
|  | | | | | | |  |
| 8. Are you able to get in and out of a car? | %Predicted FVC - upright | 30 | 0.1446 | 0.446 | 0.0573 | 0.764 |  |
|  | %Predicted FVC - supine | 25 | 0.1079 | 0.608 | 0.0547 | 0.795 |  |
|  | Total MMT | 26 | 0.5061 | 0.008* | 0.4887 | 0.011* |  |
|  | Upper MMT | 26 | 0.3720 | 0.061 | 0.2752 | 0.174 |  |
|  | Lower MMT | 26 | 0.4749 | 0.014* | 0.4282 | 0.029* |  |
|  | Six Minute Walk Distance | 30 | 0.4510 | 0.012* | 0.3196 | 0.085 |  |
|  | %Predicted 6MWD | 30 | 0.3678 | 0.046* | 0.2567 | 0.171 |  |
|  | | | | | | |  |
| 9. Are you able to squeeze a new tube of toothpaste? | %Predicted FVC - upright | 30 | 0.1093 | 0.565 | 0.1180 | 0.534 |  |
|  | %Predicted FVC - supine | 25 | 0.1942 | 0.352 | 0.1982 | 0.342 |  |
|  | Total MMT | 26 | 0.1650 | 0.421 | 0.1606 | 0.433 |  |
|  | Upper MMT | 26 | 0.1795 | 0.380 | 0.2329 | 0.252 |  |
|  | Lower MMT | 26 | 0.1196 | 0.561 | 0.1206 | 0.557 |  |
|  | Six Minute Walk Distance | 30 | 0.0684 | 0.719 | 0.0965 | 0.612 |  |
|  | %Predicted 6MWD | 30 | 0.0175 | 0.927 | -0.0322 | 0.866 |  |
|  | | | | | | |  |
| 10. Are you able to hold a plate full of food? | %Predicted FVC - upright | 30 | 0.0922 | 0.628 | -0.0137 | 0.943 |  |
|  | %Predicted FVC - supine | 25 | -0.0682 | 0.746 | -0.0971 | 0.644 |  |
|  | Total MMT | 26 | 0.5320 | 0.005* | 0.5377 | 0.005* |  |
|  | Upper MMT | 26 | 0.7120 | 0.000* | 0.5913 | 0.001* |  |
|  | Lower MMT | 26 | 0.3054 | 0.129 | 0.3120 | 0.121 |  |
|  | Six Minute Walk Distance | 30 | 0.2832 | 0.129 | 0.3323 | 0.073 |  |
|  | %Predicted 6MWD | 30 | 0.2925 | 0.117 | 0.2413 | 0.199 |  |
|  | | | | | | |  |
| 11. Are you able to run a short distance, such as to catch a bus? | %Predicted FVC - upright | 30 | 0.0551 | 0.772 | -0.0666 | 0.727 |  |
|  | %Predicted FVC - supine | 25 | 0.2023 | 0.332 | 0.2215 | 0.287 |  |
|  | Total MMT | 26 | 0.4550 | 0.020* | 0.5185 | 0.007* |  |
|  | Upper MMT | 26 | 0.3246 | 0.106 | 0.3317 | 0.098 |  |
|  | Lower MMT | 26 | 0.4330 | 0.027* | 0.4845 | 0.012* |  |
|  | Six Minute Walk Distance | 30 | 0.3914 | 0.032* | 0.3583 | 0.052 |  |
|  | %Predicted 6MWD | 30 | 0.1791 | 0.344 | 0.1684 | 0.374 |  |
|  | | | | | | |  |
| 12. Are you able to shampoo your hair? | %Predicted FVC - upright | 30 | 0.3076 | 0.098 | 0.2037 | 0.280 |  |
|  | %Predicted FVC - supine | 25 | 0.1548 | 0.460 | 0.1871 | 0.371 |  |
|  | Total MMT | 26 | 0.4748 | 0.014* | 0.4464 | 0.022* |  |
|  | Upper MMT | 26 | 0.6603 | 0.000* | 0.4397 | 0.025* |  |
|  | Lower MMT | 26 | 0.2575 | 0.204 | 0.2424 | 0.233 |  |
|  | Six Minute Walk Distance | 30 | 0.3660 | 0.047* | 0.4073 | 0.025* |  |
|  | %Predicted 6MWD | 30 | 0.3104 | 0.095 | 0.2291 | 0.223 |  |
|  | | | | | | |  |
| 13. Are you able to sit on and get up from the toilet? | %Predicted FVC - upright | 29 | -0.0022 | 0.991 | -0.0870 | 0.653 |  |
|  | %Predicted FVC - supine | 25 | 0.1229 | 0.567 | 0.1476 | 0.491 |  |
|  | Total MMT | 26 | 0.4457 | 0.026* | 0.3936 | 0.052 |  |
|  | Upper MMT | 26 | 0.5082 | 0.009* | 0.3837 | 0.058 |  |
|  | Lower MMT | 26 | 0.3075 | 0.135 | 0.3466 | 0.090 |  |
|  | Six Minute Walk Distance | 29 | 0.3257 | 0.085 | 0.3842 | 0.040* |  |
|  | %Predicted 6MWD | 29 | 0.2544 | 0.183 | 0.2413 | 0.207 |  |
|  | | | | | | |  |
| 14. Are you able to transfer from a bed to a chair and back? | %Predicted FVC - upright | 29 | 0.2423 | 0.205 | 0.1580 | 0.413 |  |
|  | %Predicted FVC - supine | 25 | 0.2954 | 0.161 | 0.3069 | 0.145 |  |
|  | Total MMT | 26 | 0.4101 | 0.042* | 0.3831 | 0.059 |  |
|  | Upper MMT | 26 | 0.4707 | 0.018* | 0.1785 | 0.393 |  |
|  | Lower MMT | 26 | 0.2810 | 0.174 | 0.2855 | 0.166 |  |
|  | Six Minute Walk Distance | 29 | 0.2288 | 0.233 | 0.2780 | 0.144 |  |
|  | %Predicted 6MWD | 29 | 0.2051 | 0.286 | 0.1541 | 0.425 |  |
|  | | | | | | |  |
| 15. Does your health now limit you in doing vigorous activities, such as running, lifting heavy objects, participating in strenuous sports? | %Predicted FVC - upright | 30 | 0.1648 | 0.384 | 0.0881 | 0.643 |  |
|  | %Predicted FVC - supine | 25 | 0.1276 | 0.543 | 0.0629 | 0.765 |  |
|  | Total MMT | 26 | 0.3568 | 0.074 | 0.2207 | 0.279 |  |
|  | Upper MMT | 26 | 0.2491 | 0.220 | 0.2177 | 0.285 |  |
|  | Lower MMT | 26 | 0.3427 | 0.087 | 0.1186 | 0.564 |  |
|  | Six Minute Walk Distance | 30 | 0.2378 | 0.206 | 0.2144 | 0.255 |  |
|  | %Predicted 6MWD | 30 | 0.0878 | 0.645 | 0.0638 | 0.738 |  |
|  | | | | | | |  |
| 16. Does your health now limit you in bending, kneeling, or stooping? | %Predicted FVC - upright | 30 | 0.0967 | 0.611 | -0.0193 | 0.919 |  |
|  | %Predicted FVC - supine | 25 | 0.1685 | 0.421 | 0.1197 | 0.569 |  |
|  | Total MMT | 26 | 0.4310 | 0.028* | 0.4581 | 0.019* |  |
|  | Upper MMT | 26 | 0.2809 | 0.165 | 0.3362 | 0.093 |  |
|  | Lower MMT | 26 | 0.4262 | 0.030* | 0.4319 | 0.028* |  |
|  | Six Minute Walk Distance | 30 | 0.3616 | 0.050* | 0.3663 | 0.046* |  |
|  | %Predicted 6MWD | 30 | 0.2290 | 0.224 | 0.2742 | 0.143 |  |
|  | | | | | | |  |
| 17. Does your health limit you in lifting or carrying groceries? | %Predicted FVC - upright | 29 | 0.0460 | 0.813 | -0.1011 | 0.602 |  |
|  | %Predicted FVC - supine | 25 | 0.0046 | 0.983 | -0.0924 | 0.667 |  |
|  | Total MMT | 26 | 0.5587 | 0.004* | 0.5507 | 0.004* |  |
|  | Upper MMT | 26 | 0.3962 | 0.050* | 0.3273 | 0.110 |  |
|  | Lower MMT | 26 | 0.5387 | 0.005* | 0.4605 | 0.021* |  |
|  | Six Minute Walk Distance | 29 | 0.4541 | 0.013* | 0.3840 | 0.040* |  |
|  | %Predicted 6MWD | 29 | 0.2918 | 0.125 | 0.3097 | 0.102 |  |
|  | | | | | | |  |
| 18. Does your health now limit you in doing two hours of physical labor? | %Predicted FVC - upright | 30 | 0.2335 | 0.214 | 0.2003 | 0.289 |  |
|  | %Predicted FVC - supine | 25 | 0.2581 | 0.213 | 0.2874 | 0.164 |  |
|  | Total MMT | 26 | 0.4848 | 0.012* | 0.4812 | 0.013* |  |
|  | Upper MMT | 26 | 0.4432 | 0.023* | 0.4532 | 0.020* |  |
|  | Lower MMT | 26 | 0.4025 | 0.041* | 0.3486 | 0.081 |  |
|  | Six Minute Walk Distance | 30 | 0.5190 | 0.003* | 0.5916 | 0.001* |  |
|  | %Predicted 6MWD | 30 | 0.3717 | 0.043* | 0.4114 | 0.024* |  |
|  | | | | | | |  |
| 19. Does your health now limit you in walking more than a mile (1.6 km)? | %Predicted FVC - upright | 30 | 0.3260 | 0.079 | 0.2473 | 0.188 |  |
|  | %Predicted FVC - supine | 25 | 0.3626 | 0.075 | 0.2657 | 0.199 |  |
|  | Total MMT | 26 | 0.6064 | 0.001* | 0.6323 | 0.001* |  |
|  | Upper MMT | 26 | 0.5082 | 0.008* | 0.5184 | 0.007* |  |
|  | Lower MMT | 26 | 0.5314 | 0.005* | 0.4878 | 0.011* |  |
|  | Six Minute Walk Distance | 30 | 0.6259 | 0.000* | 0.6129 | 0.000* |  |
|  | %Predicted 6MWD | 30 | 0.4460 | 0.013* | 0.4237 | 0.020* |  |
|  | | | | | | |  |
| 20. Does your health now limit you in climbing one flight of stairs? | %Predicted FVC - upright | 30 | 0.2016 | 0.285 | 0.1710 | 0.366 |  |
|  | %Predicted FVC - supine | 25 | 0.1921 | 0.358 | 0.2102 | 0.313 |  |
|  | Total MMT | 26 | 0.5608 | 0.003* | 0.5600 | 0.003* |  |
|  | Upper MMT | 26 | 0.4632 | 0.017* | 0.4412 | 0.024* |  |
|  | Lower MMT | 26 | 0.4954 | 0.010* | 0.4958 | 0.010* |  |
|  | Six Minute Walk Distance | 30 | 0.5738 | 0.001* | 0.5971 | 0.000* |  |
|  | %Predicted 6MWD | 30 | 0.3787 | 0.039* | 0.4363 | 0.016* |  |

|  |
| --- |

6MWD = 6-Minute Walk Distance; FVC = forced vital capacity; MMT = Manual Muscle Test.

| Table 10 | | | | | | | |
| --- | --- | --- | --- | --- | --- | --- | --- |
| Correlation between Each Dyspnea Severity Question Score vs All the Parameters | | | | | | | |
| (Patients with late-onset Pompe disease) | | | | | | | |
|  | | | Pearson Correlation | | Spearman Correlation | |  |
| Questions | Parameters | N | Coefficient | P-value | Coefficient | P-value |  |
|  | | | | | | |  |
| 1. Dressing yourself without help | %Predicted FVC - upright | 30 | -0.4139 | 0.023* | -0.2955 | 0.113 |  |
|  | %Predicted FVC - supine | 25 | -0.2706 | 0.191 | -0.2152 | 0.302 |  |
|  | Total MMT | 26 | -0.1439 | 0.483 | -0.1819 | 0.374 |  |
|  | Upper MMT | 26 | -0.0638 | 0.757 | -0.0462 | 0.823 |  |
|  | Lower MMT | 26 | -0.1604 | 0.434 | -0.1621 | 0.429 |  |
|  | Six Minute Walk Distance | 30 | -0.1360 | 0.474 | -0.1142 | 0.548 |  |
|  | %Predicted 6MWD | 30 | -0.0996 | 0.600 | -0.0144 | 0.940 |  |
|  | | | | | | |  |
| 2. Walking 50 steps/paces on flat ground at a normal speed without stopping | %Predicted FVC - upright | 29 | -0.2581 | 0.176 | -0.1818 | 0.345 |  |
|  | %Predicted FVC - supine | 25 | -0.2013 | 0.346 | -0.1502 | 0.484 |  |
|  | Total MMT | 26 | -0.1587 | 0.449 | -0.1330 | 0.526 |  |
|  | Upper MMT | 26 | -0.1003 | 0.633 | -0.0953 | 0.651 |  |
|  | Lower MMT | 26 | -0.1588 | 0.448 | -0.1103 | 0.600 |  |
|  | Six Minute Walk Distance | 29 | -0.0033 | 0.986 | -0.0328 | 0.866 |  |
|  | %Predicted 6MWD | 29 | -0.0590 | 0.761 | -0.0360 | 0.853 |  |
|  | | | | | | |  |
| 3. Walking up 20 stairs (2 flights) without stopping | %Predicted FVC - upright | 21 | -0.4142 | 0.062 | -0.2889 | 0.204 |  |
|  | %Predicted FVC - supine | 21 | -0.4911 | 0.038* | -0.4309 | 0.074 |  |
|  | Total MMT | 21 | -0.2344 | 0.349 | -0.2350 | 0.348 |  |
|  | Upper MMT | 21 | 0.0000 | 1.000 | 0.0342 | 0.893 |  |
|  | Lower MMT | 21 | -0.3131 | 0.206 | -0.2191 | 0.382 |  |
|  | Six Minute Walk Distance | 21 | -0.1092 | 0.638 | -0.1475 | 0.524 |  |
|  | %Predicted 6MWD | 21 | 0.0457 | 0.844 | 0.0437 | 0.851 |  |
|  | | | | | | |  |
| 4. Preparing meals | %Predicted FVC - upright | 28 | -0.3497 | 0.068 | -0.2533 | 0.193 |  |
|  | %Predicted FVC - supine | 25 | 0.0748 | 0.728 | 0.1001 | 0.642 |  |
|  | Total MMT | 26 | -0.2107 | 0.323 | -0.3178 | 0.130 |  |
|  | Upper MMT | 26 | -0.3805 | 0.067 | -0.3678 | 0.077 |  |
|  | Lower MMT | 26 | -0.0813 | 0.706 | -0.1054 | 0.624 |  |
|  | Six Minute Walk Distance | 28 | -0.3599 | 0.060 | -0.3711 | 0.052 |  |
|  | %Predicted 6MWD | 28 | -0.3766 | 0.048* | -0.2955 | 0.127 |  |
|  | | | | | | |  |
| 5. Washing dishes | %Predicted FVC - upright | 29 | -0.4368 | 0.018* | -0.2664 | 0.162 |  |
|  | %Predicted FVC - supine | 25 | -0.0040 | 0.985 | 0.0000 | 1.000 |  |
|  | Total MMT | 26 | -0.1120 | 0.594 | -0.1797 | 0.390 |  |
|  | Upper MMT | 26 | -0.2332 | 0.262 | -0.2191 | 0.293 |  |
|  | Lower MMT | 26 | -0.0257 | 0.903 | -0.0400 | 0.849 |  |
|  | Six Minute Walk Distance | 29 | -0.2239 | 0.243 | -0.2277 | 0.235 |  |
|  | %Predicted 6MWD | 29 | -0.2400 | 0.210 | -0.1562 | 0.418 |  |
|  | | | | | | |  |
| 6. Sweeping or mopping | %Predicted FVC - upright | 23 | -0.3499 | 0.102 | -0.2551 | 0.240 |  |
|  | %Predicted FVC - supine | 23 | -0.1909 | 0.420 | -0.1867 | 0.431 |  |
|  | Total MMT | 23 | -0.2558 | 0.276 | -0.3859 | 0.093 |  |
|  | Upper MMT | 23 | -0.1480 | 0.533 | -0.1032 | 0.665 |  |
|  | Lower MMT | 23 | -0.2567 | 0.275 | -0.3243 | 0.163 |  |
|  | Six Minute Walk Distance | 23 | -0.4435 | 0.034* | -0.3697 | 0.083 |  |
|  | %Predicted 6MWD | 23 | -0.3073 | 0.154 | -0.1105 | 0.616 |  |
|  | | | | | | |  |
| 7. Making a bed | %Predicted FVC - upright | 25 | -0.2778 | 0.179 | -0.1989 | 0.341 |  |
|  | %Predicted FVC - supine | 25 | -0.1948 | 0.385 | -0.1304 | 0.563 |  |
|  | Total MMT | 25 | -0.2219 | 0.321 | -0.3385 | 0.123 |  |
|  | Upper MMT | 25 | -0.2963 | 0.181 | -0.3166 | 0.151 |  |
|  | Lower MMT | 25 | -0.1420 | 0.528 | -0.1723 | 0.443 |  |
|  | Six Minute Walk Distance | 25 | -0.1259 | 0.549 | -0.1251 | 0.551 |  |
|  | %Predicted 6MWD | 25 | -0.2228 | 0.284 | -0.1282 | 0.542 |  |
|  | | | | | | |  |
| 8. Lifting something weighing 10-20 lbs (about 4.5-9kg, like a large bag of groceries) | %Predicted FVC - upright | 23 | -0.3407 | 0.112 | -0.2377 | 0.275 |  |
|  | %Predicted FVC - supine | 23 | -0.2593 | 0.284 | -0.2326 | 0.338 |  |
|  | Total MMT | 23 | -0.3288 | 0.157 | -0.3624 | 0.116 |  |
|  | Upper MMT | 23 | -0.3381 | 0.145 | -0.3249 | 0.162 |  |
|  | Lower MMT | 23 | -0.2497 | 0.288 | -0.2370 | 0.314 |  |
|  | Six Minute Walk Distance | 23 | -0.4173 | 0.048* | -0.4238 | 0.044* |  |
|  | %Predicted 6MWD | 23 | -0.4230 | 0.044* | -0.3976 | 0.060 |  |
|  | | | | | | |  |
| 9. Carrying something weighing 10-20 lbs (about 4.5-9kg, like a large bag of groceries) from one room to another | %Predicted FVC - upright | 22 | -0.3106 | 0.160 | -0.2287 | 0.306 |  |
|  | %Predicted FVC - supine | 22 | -0.2547 | 0.293 | -0.2508 | 0.300 |  |
|  | Total MMT | 22 | -0.5343 | 0.018* | -0.5587 | 0.013* |  |
|  | Upper MMT | 22 | -0.5427 | 0.016* | -0.5291 | 0.020* |  |
|  | Lower MMT | 22 | -0.4218 | 0.072 | -0.4002 | 0.090 |  |
|  | Six Minute Walk Distance | 22 | -0.4453 | 0.038* | -0.3948 | 0.069 |  |
|  | %Predicted 6MWD | 22 | -0.3788 | 0.082 | -0.3111 | 0.159 |  |
|  | | | | | | |  |
| 10. Walking (faster than your usual speed) for 1/2 mile (almost 1 km) without stopping | %Predicted FVC - upright | 19 | -0.2657 | 0.272 | -0.1632 | 0.504 |  |
|  | %Predicted FVC - supine | 19 | -0.3912 | 0.134 | -0.2772 | 0.299 |  |
|  | Total MMT | 19 | -0.4905 | 0.054 | -0.5131 | 0.042* |  |
|  | Upper MMT | 19 | -0.4620 | 0.072 | -0.5338 | 0.033* |  |
|  | Lower MMT | 19 | -0.4314 | 0.095 | -0.4043 | 0.120 |  |
|  | Six Minute Walk Distance | 19 | -0.4507 | 0.053 | -0.4404 | 0.059 |  |
|  | %Predicted 6MWD | 19 | -0.3900 | 0.099 | -0.3767 | 0.112 |  |
|  | | | | | | |  |
| 11. Taking a bath without help | %Predicted FVC - upright | 22 | -0.5889 | 0.004* | -0.4396 | 0.041* |  |
|  | %Predicted FVC - supine | 22 | -0.2788 | 0.279 | -0.2364 | 0.361 |  |
|  | Total MMT | 22 | 0.0269 | 0.913 | -0.0199 | 0.936 |  |
|  | Upper MMT | 22 | 0.0395 | 0.872 | 0.0079 | 0.975 |  |
|  | Lower MMT | 22 | 0.0141 | 0.954 | 0.0624 | 0.800 |  |
|  | Six Minute Walk Distance | 22 | -0.2157 | 0.335 | -0.2737 | 0.218 |  |
|  | %Predicted 6MWD | 22 | -0.0933 | 0.680 | 0.0771 | 0.733 |  |
|  | | | | | | |  |
| 12. Taking a shower | %Predicted FVC - upright | 30 | -0.5464 | 0.002* | -0.4432 | 0.014* |  |
|  | %Predicted FVC - supine | 25 | -0.3064 | 0.136 | -0.2923 | 0.156 |  |
|  | Total MMT | 26 | 0.0058 | 0.978 | 0.0031 | 0.988 |  |
|  | Upper MMT | 26 | -0.0531 | 0.797 | -0.0212 | 0.918 |  |
|  | Lower MMT | 26 | 0.0400 | 0.846 | 0.0989 | 0.631 |  |
|  | Six Minute Walk Distance | 30 | -0.1994 | 0.291 | -0.2140 | 0.256 |  |
|  | %Predicted 6MWD | 30 | -0.1322 | 0.486 | -0.0242 | 0.899 |  |
|  | | | | | | |  |
| 13. Putting on socks or stockings | %Predicted FVC - upright | 30 | -0.3182 | 0.087 | -0.2945 | 0.114 |  |
|  | %Predicted FVC - supine | 25 | -0.2840 | 0.169 | -0.2375 | 0.253 |  |
|  | Total MMT | 26 | -0.1041 | 0.613 | -0.1294 | 0.529 |  |
|  | Upper MMT | 26 | -0.1468 | 0.474 | -0.0898 | 0.663 |  |
|  | Lower MMT | 26 | -0.0553 | 0.789 | -0.0448 | 0.828 |  |
|  | Six Minute Walk Distance | 30 | -0.0292 | 0.878 | -0.0075 | 0.969 |  |
|  | %Predicted 6MWD | 30 | 0.0031 | 0.987 | 0.0685 | 0.719 |  |
|  | | | | | | |  |
| 14. Standing for at least 5 minutes | %Predicted FVC - upright | 30 | -0.3936 | 0.031* | -0.3076 | 0.098 |  |
|  | %Predicted FVC - supine | 25 | -0.1282 | 0.541 | -0.1400 | 0.504 |  |
|  | Total MMT | 26 | -0.2223 | 0.275 | -0.1652 | 0.420 |  |
|  | Upper MMT | 26 | -0.3222 | 0.108 | -0.2815 | 0.164 |  |
|  | Lower MMT | 26 | -0.1127 | 0.584 | -0.0441 | 0.831 |  |
|  | Six Minute Walk Distance | 30 | -0.2102 | 0.265 | -0.2726 | 0.145 |  |
|  | %Predicted 6MWD | 30 | -0.2693 | 0.150 | -0.2606 | 0.164 |  |
|  | | | | | | |  |
| 15. Walking 10 steps/paces on flat ground at a normal speed without stopping | %Predicted FVC - upright | 29 | -0.5074 | 0.005* | -0.4254 | 0.021* |  |
|  | %Predicted FVC - supine | 25 | -0.2113 | 0.322 | -0.1986 | 0.352 |  |
|  | Total MMT | 26 | -0.1854 | 0.375 | -0.2957 | 0.151 |  |
|  | Upper MMT | 26 | -0.2541 | 0.220 | -0.3891 | 0.055 |  |
|  | Lower MMT | 26 | -0.1008 | 0.632 | -0.1350 | 0.520 |  |
|  | Six Minute Walk Distance | 29 | -0.5411 | 0.002* | -0.5544 | 0.002* |  |
|  | %Predicted 6MWD | 29 | -0.4640 | 0.011* | -0.4373 | 0.018* |  |
|  | | | | | | |  |
| 16. Walking 1/2 mile (almost 1 km) on flat ground at a normal speed without stopping | %Predicted FVC - upright | 20 | -0.6370 | 0.003* | -0.5872 | 0.006* |  |
|  | %Predicted FVC - supine | 20 | -0.7975 | 0.000* | -0.8160 | 0.000* |  |
|  | Total MMT | 20 | -0.1515 | 0.562 | -0.1412 | 0.589 |  |
|  | Upper MMT | 20 | -0.2470 | 0.339 | -0.2291 | 0.376 |  |
|  | Lower MMT | 20 | -0.0525 | 0.841 | -0.0370 | 0.888 |  |
|  | Six Minute Walk Distance | 20 | -0.4761 | 0.034* | -0.5018 | 0.024* |  |
|  | %Predicted 6MWD | 20 | -0.4169 | 0.067 | -0.3896 | 0.090 |  |
|  | | | | | | |  |
| 17. Walking up 5 stairs without stopping | %Predicted FVC - upright | 27 | -0.3601 | 0.065 | -0.2418 | 0.224 |  |
|  | %Predicted FVC - supine | 25 | -0.2707 | 0.223 | -0.3036 | 0.170 |  |
|  | Total MMT | 26 | -0.2602 | 0.230 | -0.2866 | 0.185 |  |
|  | Upper MMT | 26 | -0.3696 | 0.083 | -0.3396 | 0.113 |  |
|  | Lower MMT | 26 | -0.1332 | 0.545 | -0.1755 | 0.423 |  |
|  | Six Minute Walk Distance | 27 | -0.3178 | 0.106 | -0.3554 | 0.069 |  |
|  | %Predicted 6MWD | 27 | -0.3063 | 0.120 | -0.2768 | 0.162 |  |
|  | | | | | | |  |
| 18. Walking up 10 stairs (1 flight) without stopping | %Predicted FVC - upright | 22 | -0.3572 | 0.103 | -0.2592 | 0.244 |  |
|  | %Predicted FVC - supine | 22 | -0.3061 | 0.217 | -0.2550 | 0.307 |  |
|  | Total MMT | 22 | -0.4824 | 0.043* | -0.4724 | 0.048* |  |
|  | Upper MMT | 22 | -0.3119 | 0.208 | -0.2553 | 0.307 |  |
|  | Lower MMT | 22 | -0.4860 | 0.041* | -0.4062 | 0.094 |  |
|  | Six Minute Walk Distance | 22 | -0.4401 | 0.040* | -0.3746 | 0.086 |  |
|  | %Predicted 6MWD | 22 | -0.2105 | 0.347 | -0.1319 | 0.558 |  |
|  | | | | | | |  |
| 19. Walking up 30 stairs (3 flights) without stopping | %Predicted FVC - upright | 14 | -0.4933 | 0.073 | -0.2871 | 0.320 |  |
|  | %Predicted FVC - supine | 14 | -0.4484 | 0.144 | -0.2151 | 0.502 |  |
|  | Total MMT | 14 | -0.4813 | 0.113 | -0.3428 | 0.275 |  |
|  | Upper MMT | 14 | -0.2657 | 0.404 | -0.1503 | 0.641 |  |
|  | Lower MMT | 14 | -0.4823 | 0.112 | -0.2938 | 0.354 |  |
|  | Six Minute Walk Distance | 14 | -0.2671 | 0.356 | -0.2289 | 0.431 |  |
|  | %Predicted 6MWD | 14 | -0.0939 | 0.749 | -0.1734 | 0.553 |  |
|  | | | | | | |  |
| 20. Lifting something weighing less than 5 lbs (about 2 kg, like a houseplant) | %Predicted FVC - upright | 30 | -0.2393 | 0.203 | -0.0363 | 0.849 |  |
|  | %Predicted FVC - supine | 25 | -0.1095 | 0.602 | -0.1050 | 0.617 |  |
|  | Total MMT | 26 | -0.2130 | 0.296 | -0.1952 | 0.339 |  |
|  | Upper MMT | 26 | -0.3301 | 0.100 | -0.1941 | 0.342 |  |
|  | Lower MMT | 26 | -0.0950 | 0.644 | -0.0614 | 0.766 |  |
|  | Six Minute Walk Distance | 30 | -0.1551 | 0.413 | -0.2196 | 0.244 |  |
|  | %Predicted 6MWD | 30 | -0.0697 | 0.714 | -0.0078 | 0.967 |  |
|  | | | | | | |  |
| 21. Lifting something weighing 5-10 lbs (about 2-4.5 kg, like a basket of clothes) | %Predicted FVC - upright | 28 | -0.2069 | 0.291 | -0.0275 | 0.890 |  |
|  | %Predicted FVC - supine | 25 | -0.0531 | 0.810 | -0.0971 | 0.659 |  |
|  | Total MMT | 26 | -0.3748 | 0.071 | -0.3961 | 0.055 |  |
|  | Upper MMT | 26 | -0.4530 | 0.026* | -0.3587 | 0.085 |  |
|  | Lower MMT | 26 | -0.2434 | 0.252 | -0.2828 | 0.181 |  |
|  | Six Minute Walk Distance | 28 | -0.1810 | 0.357 | -0.3032 | 0.117 |  |
|  | %Predicted 6MWD | 28 | -0.1276 | 0.518 | -0.1341 | 0.496 |  |
|  | | | | | | |  |
| 22. Lifting something weighing more than 20 lbs (about 9 kg, like a medium-sized suitcase) | %Predicted FVC - upright | 20 | -0.0141 | 0.953 | 0.0727 | 0.761 |  |
|  | %Predicted FVC - supine | 20 | -0.0669 | 0.799 | -0.0384 | 0.884 |  |
|  | Total MMT | 20 | -0.4227 | 0.091 | -0.3763 | 0.137 |  |
|  | Upper MMT | 20 | -0.4608 | 0.063 | -0.3146 | 0.219 |  |
|  | Lower MMT | 20 | -0.3322 | 0.193 | -0.3391 | 0.183 |  |
|  | Six Minute Walk Distance | 20 | 0.1012 | 0.671 | 0.0039 | 0.987 |  |
|  | %Predicted 6MWD | 20 | 0.1688 | 0.477 | 0.2283 | 0.333 |  |
|  | | | | | | |  |
| 23. Carrying something weighing less than 5 lbs (about 2 kg, like a houseplant) from one room to another | %Predicted FVC - upright | 29 | -0.0949 | 0.624 | 0.0827 | 0.670 |  |
|  | %Predicted FVC - supine | 25 | 0.0940 | 0.662 | 0.1099 | 0.609 |  |
|  | Total MMT | 26 | -0.2458 | 0.236 | -0.2735 | 0.186 |  |
|  | Upper MMT | 26 | -0.1228 | 0.559 | -0.0842 | 0.689 |  |
|  | Lower MMT | 26 | -0.2697 | 0.192 | -0.2983 | 0.147 |  |
|  | Six Minute Walk Distance | 29 | -0.1619 | 0.402 | -0.2069 | 0.281 |  |
|  | %Predicted 6MWD | 29 | -0.0134 | 0.945 | -0.0312 | 0.872 |  |
|  | | | | | | |  |
| 24. Carrying something weighing 5-10 lbs (about 2-4.5 kg, like a basket of clothes) from one room to another | %Predicted FVC - upright | 26 | -0.1355 | 0.509 | 0.0370 | 0.858 |  |
|  | %Predicted FVC - supine | 25 | -0.0746 | 0.748 | -0.0797 | 0.731 |  |
|  | Total MMT | 26 | -0.4504 | 0.035* | -0.4431 | 0.039* |  |
|  | Upper MMT | 26 | -0.2637 | 0.236 | -0.1472 | 0.513 |  |
|  | Lower MMT | 26 | -0.4677 | 0.028* | -0.4907 | 0.020* |  |
|  | Six Minute Walk Distance | 26 | -0.2264 | 0.266 | -0.2840 | 0.160 |  |
|  | %Predicted 6MWD | 26 | -0.0732 | 0.722 | -0.0380 | 0.854 |  |
|  | | | | | | |  |
| 25. Getting in or out of a car | %Predicted FVC - upright | 29 | -0.4047 | 0.029* | -0.3833 | 0.040* |  |
|  | %Predicted FVC - supine | 25 | -0.3802 | 0.067 | -0.3409 | 0.103 |  |
|  | Total MMT | 26 | -0.1482 | 0.480 | -0.1098 | 0.601 |  |
|  | Upper MMT | 26 | -0.0612 | 0.771 | 0.0188 | 0.929 |  |
|  | Lower MMT | 26 | -0.1708 | 0.414 | -0.1101 | 0.600 |  |
|  | Six Minute Walk Distance | 29 | -0.2807 | 0.140 | -0.3221 | 0.088 |  |
|  | %Predicted 6MWD | 29 | -0.1549 | 0.422 | -0.1050 | 0.588 |  |
|  | | | | | | |  |
| 26. Dining out | %Predicted FVC - upright | 29 | -0.4679 | 0.010* | -0.4999 | 0.006* |  |
|  | %Predicted FVC - supine | 25 | -0.3346 | 0.110 | -0.3566 | 0.087 |  |
|  | Total MMT | 26 | -0.1853 | 0.375 | -0.1465 | 0.485 |  |
|  | Upper MMT | 26 | -0.2964 | 0.150 | -0.3079 | 0.134 |  |
|  | Lower MMT | 26 | -0.0738 | 0.726 | -0.0121 | 0.954 |  |
|  | Six Minute Walk Distance | 29 | -0.1637 | 0.396 | -0.1880 | 0.329 |  |
|  | %Predicted 6MWD | 29 | -0.2216 | 0.248 | -0.2187 | 0.254 |  |
|  | | | | | | |  |
| 27. Low-intensity leisure activity (gardening, etc.) | %Predicted FVC - upright | 23 | -0.6243 | 0.001* | -0.6253 | 0.001* |  |
|  | %Predicted FVC - supine | 23 | -0.4956 | 0.036* | -0.4614 | 0.054 |  |
|  | Total MMT | 23 | -0.3055 | 0.203 | -0.3263 | 0.173 |  |
|  | Upper MMT | 23 | -0.1439 | 0.557 | -0.0363 | 0.883 |  |
|  | Lower MMT | 23 | -0.3553 | 0.135 | -0.3148 | 0.189 |  |
|  | Six Minute Walk Distance | 23 | -0.3433 | 0.109 | -0.3940 | 0.063 |  |
|  | %Predicted 6MWD | 23 | -0.3795 | 0.074 | -0.3362 | 0.117 |  |
|  | | | | | | |  |
| 28. Moderate-intensity leisure activity (bicycling on level terrain, etc.) | %Predicted FVC - upright | 18 | -0.5846 | 0.011* | -0.6167 | 0.006* |  |
|  | %Predicted FVC - supine | 18 | -0.5339 | 0.040* | -0.5697 | 0.027* |  |
|  | Total MMT | 18 | -0.3227 | 0.223 | -0.3690 | 0.160 |  |
|  | Upper MMT | 18 | -0.1831 | 0.497 | -0.1329 | 0.624 |  |
|  | Lower MMT | 18 | -0.3683 | 0.160 | -0.3524 | 0.181 |  |
|  | Six Minute Walk Distance | 18 | -0.2115 | 0.400 | -0.2935 | 0.237 |  |
|  | %Predicted 6MWD | 18 | -0.3110 | 0.209 | -0.2738 | 0.272 |  |
|  | | | | | | |  |
| 29. Walking (faster than your usual speed) for 50 steps without stopping | %Predicted FVC - upright | 20 | -0.2513 | 0.285 | -0.2050 | 0.386 |  |
|  | %Predicted FVC - supine | 20 | -0.2639 | 0.306 | -0.2094 | 0.420 |  |
|  | Total MMT | 20 | -0.3466 | 0.159 | -0.3949 | 0.105 |  |
|  | Upper MMT | 20 | -0.2370 | 0.344 | -0.1433 | 0.570 |  |
|  | Lower MMT | 20 | -0.3655 | 0.136 | -0.3599 | 0.142 |  |
|  | Six Minute Walk Distance | 20 | -0.1683 | 0.478 | -0.1226 | 0.607 |  |
|  | %Predicted 6MWD | 20 | -0.1318 | 0.580 | -0.1155 | 0.628 |  |
|  | | | | | | |  |
| 30. Walking (faster than your usual speed) for at least 1 mile (a little more than 1.5 km) without stopping | %Predicted FVC - upright | 14 | -0.5696 | 0.033* | -0.4883 | 0.076 |  |
|  | %Predicted FVC - supine | 14 | -0.5505 | 0.064 | -0.5501 | 0.064 |  |
|  | Total MMT | 14 | -0.1288 | 0.675 | -0.1277 | 0.678 |  |
|  | Upper MMT | 14 | -0.3897 | 0.188 | -0.2818 | 0.351 |  |
|  | Lower MMT | 14 | -0.0327 | 0.916 | 0.0000 | 1.000 |  |
|  | Six Minute Walk Distance | 14 | -0.3192 | 0.266 | -0.3660 | 0.198 |  |
|  | %Predicted 6MWD | 14 | -0.4218 | 0.133 | -0.4163 | 0.139 |  |
|  | | | | | | |  |
| 31. Singing or humming | %Predicted FVC - upright | 26 | -0.1344 | 0.513 | -0.0896 | 0.663 |  |
|  | %Predicted FVC - supine | 25 | -0.2666 | 0.243 | -0.2531 | 0.268 |  |
|  | Total MMT | 26 | -0.0747 | 0.735 | -0.0659 | 0.765 |  |
|  | Upper MMT | 26 | 0.1704 | 0.437 | 0.1326 | 0.546 |  |
|  | Lower MMT | 26 | -0.2098 | 0.337 | -0.1585 | 0.470 |  |
|  | Six Minute Walk Distance | 26 | 0.1587 | 0.439 | 0.0989 | 0.631 |  |
|  | %Predicted 6MWD | 26 | 0.2220 | 0.276 | 0.2586 | 0.202 |  |
|  | | | | | | |  |
| 32. Talking while walking | %Predicted FVC - upright | 28 | -0.0907 | 0.646 | 0.0265 | 0.894 |  |
|  | %Predicted FVC - supine | 25 | -0.0688 | 0.755 | -0.0401 | 0.856 |  |
|  | Total MMT | 26 | -0.1981 | 0.353 | -0.1829 | 0.392 |  |
|  | Upper MMT | 26 | -0.1578 | 0.461 | -0.1406 | 0.512 |  |
|  | Lower MMT | 26 | -0.1817 | 0.396 | -0.1114 | 0.604 |  |
|  | Six Minute Walk Distance | 28 | -0.3189 | 0.098 | -0.3400 | 0.077 |  |
|  | %Predicted 6MWD | 28 | -0.3616 | 0.059 | -0.2817 | 0.146 |  |
|  | | | | | | |  |
| 33. Scrubbing the floor or counter | %Predicted FVC - upright | 25 | -0.5079 | 0.010* | -0.3508 | 0.086 |  |
|  | %Predicted FVC - supine | 25 | -0.2759 | 0.226 | -0.2444 | 0.286 |  |
|  | Total MMT | 25 | 0.0167 | 0.943 | -0.0341 | 0.883 |  |
|  | Upper MMT | 25 | -0.0030 | 0.990 | 0.0400 | 0.863 |  |
|  | Lower MMT | 25 | 0.0248 | 0.915 | 0.0492 | 0.832 |  |
|  | Six Minute Walk Distance | 25 | -0.0230 | 0.913 | -0.0652 | 0.757 |  |
|  | %Predicted 6MWD | 25 | -0.0542 | 0.797 | 0.0055 | 0.979 |  |

|  |
| --- |
|  |
|  |

6MWD = 6-Minute Walk Distance; FVC = forced vital capacity; MMT = Manual Muscle Test.
