# Supplementary material for: Use of the patient-reported outcomes measurement information system (PROMIS®) to assess late-onset Pompe disease severity
Source: J Patient Rep Outcomes. 2020 Oct 9;4:83. doi: 10.1186/s41687-020-00245-2 (PMC7547055; doi:10.1186/s41687-020-00245-2)
Supplement: Supplementary file 2 — Additional file 2. [file 41687_2020_245_MOESM2_ESM.zip › T1_1_baseline_Male.rtf]

Parameter	N	Mean	Standard
Deviation	Median	Min	Max	
	
Age	12	51.50	16.660	51.00	18	79	
	
Average age at diagnosis	12	45.92	17.697	43.50	17	77	
	
Average years of disease from diagnosis to the date of questionnaire	12	5.58	4.358	4.50	1	15	
	
Average years on ERT	11	5.45	3.503	5.00	1	12	
	
Average age at onset of symptoms - Muscle	11	31.91	11.726	33.00	15	50	
	
Average years from onset of muscle symptoms	11	18.36	16.176	16.00	3	59	
	
Average age at onset of symptoms - Respiratory	7	45.86	11.305	44.00	29	62	
	
Average years from onset of respiratory symptoms	7	6.86	4.525	5.00	3	16	
