# Supplementary material for: Use of the patient-reported outcomes measurement information system (PROMIS®) to assess late-onset Pompe disease severity
Source: J Patient Rep Outcomes. 2020 Oct 9;4:83. doi: 10.1186/s41687-020-00245-2 (PMC7547055; doi:10.1186/s41687-020-00245-2)
Supplement: Supplementary file 2 — Additional file 2. [file 41687_2020_245_MOESM2_ESM.zip › T1_2_baseline_Female.rtf]

Parameter	N	Mean	Standard
Deviation	Median	Min	Max	
	
Age	18	51.00	14.418	52.50	24	73	
	
Average age at diagnosis	18	43.22	14.526	46.50	13	71	
	
Average years of disease from diagnosis to the date of questionnaire	18	7.78	6.394	5.50	2	24	
	
Average years on ERT	17	6.12	3.903	6.00	1	12	
	
Average age at onset of symptoms - Muscle	17	30.90	14.614	35.00	1.25	52	
	
Average years from onset of muscle symptoms	17	19.81	14.297	15.00	3	58	
	
Average age at onset of symptoms - Respiratory	16	41.44	12.242	46.00	19	57	
	
Average years from onset of respiratory symptoms	16	9.94	11.733	8.00	1	48	
