# Supplementary material for: Use of the patient-reported outcomes measurement information system (PROMIS®) to assess late-onset Pompe disease severity
Source: J Patient Rep Outcomes. 2020 Oct 9;4:83. doi: 10.1186/s41687-020-00245-2 (PMC7547055; doi:10.1186/s41687-020-00245-2)
Supplement: Supplementary file 2 — Additional file 2. [file 41687_2020_245_MOESM2_ESM.zip › T1_4_baseline_le_Median_PP6MWD.rtf]

Parameter	N	Mean	Standard
Deviation	Median	Min	Max	
	
Age	15	49.60	19.489	49.00	18	79	
	
Average age at diagnosis	15	42.80	19.724	42.00	13	77	
	
Average years of disease from diagnosis to the date of questionnaire	15	6.80	6.625	3.00	1	24	
	
Average years on ERT	14	5.71	4.103	4.00	1	12	
	
Average age at onset of symptoms - Muscle	14	28.80	15.182	29.50	1.25	52	
	
Average years from onset of muscle symptoms	14	19.70	18.424	13.50	3	59	
	
Average age at onset of symptoms - Respiratory	11	37.64	14.229	39.00	19	62	
	
Average years from onset of respiratory symptoms	11	10.91	13.989	5.00	1	48	
