# Supplementary material for: Use of the patient-reported outcomes measurement information system (PROMIS®) to assess late-onset Pompe disease severity
Source: J Patient Rep Outcomes. 2020 Oct 9;4:83. doi: 10.1186/s41687-020-00245-2 (PMC7547055; doi:10.1186/s41687-020-00245-2)
Supplement: Supplementary file 2 — Additional file 2. [file 41687_2020_245_MOESM2_ESM.zip › T2_1_composite_scores_Male.rtf]

Parameter	N	Mean	Standard
Deviation	Median	Min	Max	
	
%Predicted FVC - Sitting	12	59.87	20.741	54.85	35	109.2	
	
%Predicted FVC - Supine	10	42.47	23.721	34.00	19	93.1	
	
Six Minute Walk Distance	12	372.18	116.789	386.07	183.61	527.06	
	
% Predicted Six Minute Walk Distance	12	57.89	18.944	54.81	31.4	87.34	
	
Total MMT Score	10	69.40	6.931	71.50	59	80	
	
Total Upper Extremity MMT	10	37.60	3.502	40.00	32	40	
	
Total Lower Extremity MMT	10	31.80	4.050	31.50	27	40	
