# Supplementary material for: Use of the patient-reported outcomes measurement information system (PROMIS®) to assess late-onset Pompe disease severity
Source: J Patient Rep Outcomes. 2020 Oct 9;4:83. doi: 10.1186/s41687-020-00245-2 (PMC7547055; doi:10.1186/s41687-020-00245-2)
Supplement: Supplementary file 2 — Additional file 2. [file 41687_2020_245_MOESM2_ESM.zip › T2_2_composite_scores_Female.rtf]

Parameter	N	Mean	Standard
Deviation	Median	Min	Max	
	
%Predicted FVC - Sitting	18	64.73	25.246	71.00	11	113	
	
%Predicted FVC - Supine	15	54.89	24.305	61.20	21	108	
	
Six Minute Walk Distance	18	351.87	113.117	379.91	92.11	513.32	
	
% Predicted Six Minute Walk Distance	18	64.37	20.803	59.23	23.18	103.13	
	
Total MMT Score	16	68.25	5.170	68.00	62	80	
	
Total Upper Extremity MMT	16	38.63	1.708	39.50	36	40	
	
Total Lower Extremity MMT	16	29.63	4.193	28.50	24	40	
