# Supplementary material for: Use of the patient-reported outcomes measurement information system (PROMIS®) to assess late-onset Pompe disease severity
Source: J Patient Rep Outcomes. 2020 Oct 9;4:83. doi: 10.1186/s41687-020-00245-2 (PMC7547055; doi:10.1186/s41687-020-00245-2)
Supplement: Supplementary file 2 — Additional file 2. [file 41687_2020_245_MOESM2_ESM.zip › T2_3_composite_scores_gt_Median_PP6MWD.rtf]

Parameter	N	Mean	Standard
Deviation	Median	Min	Max	
	
%Predicted FVC - Sitting	15	68.03	21.228	71.00	41	113	
	
%Predicted FVC - Supine	14	53.90	25.439	59.10	19	108	
	
Six Minute Walk Distance	15	441.69	60.568	436.72	319.03	527.06	
	
% Predicted Six Minute Walk Distance	15	78.45	11.292	80.51	58.54	103.13	
	
Total MMT Score	14	70.79	4.807	71.50	62	80	
	
Total Upper Extremity MMT	14	39.14	1.460	40.00	36	40	
	
Total Lower Extremity MMT	14	31.64	3.954	31.50	26	40	
