# Supplementary material for: Use of the patient-reported outcomes measurement information system (PROMIS®) to assess late-onset Pompe disease severity
Source: J Patient Rep Outcomes. 2020 Oct 9;4:83. doi: 10.1186/s41687-020-00245-2 (PMC7547055; doi:10.1186/s41687-020-00245-2)
Supplement: Supplementary file 2 — Additional file 2. [file 41687_2020_245_MOESM2_ESM.zip › T2_4_composite_scores_le_Median_PP6MWD.rtf]

Parameter	N	Mean	Standard
Deviation	Median	Min	Max	
	
%Predicted FVC - Sitting	15	57.55	24.793	53.00	11	109.2	
	
%Predicted FVC - Supine	11	44.86	23.118	39.00	21	93.1	
	
Six Minute Walk Distance	15	278.30	92.175	279.87	92.11	417.1	
	
% Predicted Six Minute Walk Distance	15	45.10	10.287	49.53	23.18	55.61	
	
Total MMT Score	12	66.25	6.092	64.50	59	80	
	
Total Upper Extremity MMT	12	37.17	3.129	38.00	32	40	
	
Total Lower Extremity MMT	12	29.08	4.209	28.00	24	40	
