# Supplementary material for: Use of the patient-reported outcomes measurement information system (PROMIS®) to assess late-onset Pompe disease severity
Source: J Patient Rep Outcomes. 2020 Oct 9;4:83. doi: 10.1186/s41687-020-00245-2 (PMC7547055; doi:10.1186/s41687-020-00245-2)
Supplement: Supplementary file 2 — Additional file 2. [file 41687_2020_245_MOESM2_ESM.zip › T3_1_1_Average_Raw_score_Promis_Male.rtf]

Parameter	N	Mean	Standard
Deviation	Median	Min	Max	
	
Pain Interference	11	18.00	9.889	18.00	8	32	
	
Fatigue	11	18.64	8.465	18.00	8	40	
	
Upper Extremity	12	27.00	8.301	30.00	13	35	
	
Physical Function	12	75.58	17.464	75.50	44	100	
	
Dyspnea	12	20.21	16.708	13.25	0	55	
