# Supplementary material for: Use of the patient-reported outcomes measurement information system (PROMIS®) to assess late-onset Pompe disease severity
Source: J Patient Rep Outcomes. 2020 Oct 9;4:83. doi: 10.1186/s41687-020-00245-2 (PMC7547055; doi:10.1186/s41687-020-00245-2)
Supplement: Supplementary file 2 — Additional file 2. [file 41687_2020_245_MOESM2_ESM.zip › T3_1_2_Average_Raw_score_Promis_Female.rtf]

Parameter	N	Mean	Standard
Deviation	Median	Min	Max	
	
Pain Interference	18	15.94	8.921	15.50	8	35	
	
Fatigue	18	26.44	7.563	27.00	12	37	
	
Upper Extremity	18	23.83	6.243	24.50	15	35	
	
Physical Function	18	68.72	10.283	70.00	47	87	
	
Dyspnea	18	28.13	20.373	26.85	0	67.6	
