# Supplementary material for: Use of the patient-reported outcomes measurement information system (PROMIS®) to assess late-onset Pompe disease severity
Source: J Patient Rep Outcomes. 2020 Oct 9;4:83. doi: 10.1186/s41687-020-00245-2 (PMC7547055; doi:10.1186/s41687-020-00245-2)
Supplement: Supplementary file 2 — Additional file 2. [file 41687_2020_245_MOESM2_ESM.zip › T3_1_3_Average_Raw_score_Promis_gt_Median_PP6MWD.rtf]

Parameter	N	Mean	Standard
Deviation	Median	Min	Max	
	
Pain Interference	15	15.33	8.200	15.00	8	32	
	
Fatigue	15	23.73	7.450	23.00	12	40	
	
Upper Extremity	15	28.60	5.998	30.00	17	35	
	
Physical Function	15	74.07	13.296	72.00	47	98	
	
Dyspnea	15	23.15	16.045	21.20	2.8	50.1	
