# Supplementary material for: Use of the patient-reported outcomes measurement information system (PROMIS®) to assess late-onset Pompe disease severity
Source: J Patient Rep Outcomes. 2020 Oct 9;4:83. doi: 10.1186/s41687-020-00245-2 (PMC7547055; doi:10.1186/s41687-020-00245-2)
Supplement: Supplementary file 2 — Additional file 2. [file 41687_2020_245_MOESM2_ESM.zip › T3_1_4_Average_Raw_score_Promis_le_Median_PP6MWD.rtf]

Parameter	N	Mean	Standard
Deviation	Median	Min	Max	
	
Pain Interference	14	18.21	10.222	18.50	8	35	
	
Fatigue	14	23.21	10.101	20.50	8	37	
	
Upper Extremity	15	21.60	6.663	21.00	13	35	
	
Physical Function	15	68.87	14.177	70.00	44	100	
	
Dyspnea	15	26.77	22.161	23.60	0	67.6	
