# Supplementary material for: Use of the patient-reported outcomes measurement information system (PROMIS®) to assess late-onset Pompe disease severity
Source: J Patient Rep Outcomes. 2020 Oct 9;4:83. doi: 10.1186/s41687-020-00245-2 (PMC7547055; doi:10.1186/s41687-020-00245-2)
Supplement: Supplementary file 2 — Additional file 2. [file 41687_2020_245_MOESM2_ESM.zip › T3_1_Average_Raw_score_Promis.rtf]

Parameter	N	Mean	Standard
Deviation	Median	Min	Max	
	
Pain Interference	29	16.72	9.180	16.00	8	35	
	
Fatigue	29	23.48	8.671	22.00	8	40	
	
Upper Extremity	30	25.10	7.174	25.00	13	35	
	
Physical Function	30	71.47	13.761	70.50	44	100	
	
Dyspnea	30	24.96	19.099	22.80	0	67.6	
