# Supplementary material for: Use of the patient-reported outcomes measurement information system (PROMIS®) to assess late-onset Pompe disease severity
Source: J Patient Rep Outcomes. 2020 Oct 9;4:83. doi: 10.1186/s41687-020-00245-2 (PMC7547055; doi:10.1186/s41687-020-00245-2)
Supplement: Supplementary file 2 — Additional file 2. [file 41687_2020_245_MOESM2_ESM.zip › T3_2_1_Average_T_score_Promis_Male.rtf]

Parameter	N	Mean	Standard
Deviation	Median	Min	Max	
	
Pain Interference	11	53.60	11.171	57.40	40.7	66.9	
	
Fatigue	11	51.45	11.081	51.50	33.1	77.8	
	
Upper Extremity	12	42.46	11.855	42.35	26.6	58.2	
	
Physical Function	12	41.31	10.648	39.10	26.2	62.7	
	
Dyspnea	12	37.55	9.061	35.20	28.3	60.7	
