# Supplementary material for: Use of the patient-reported outcomes measurement information system (PROMIS®) to assess late-onset Pompe disease severity
Source: J Patient Rep Outcomes. 2020 Oct 9;4:83. doi: 10.1186/s41687-020-00245-2 (PMC7547055; doi:10.1186/s41687-020-00245-2)
Supplement: Supplementary file 2 — Additional file 2. [file 41687_2020_245_MOESM2_ESM.zip › T3_2_2_Average_T_score_Promis_Female.rtf]

Parameter	N	Mean	Standard
Deviation	Median	Min	Max	
	
Pain Interference	18	51.56	10.616	55.40	40.7	69.2	
	
Fatigue	18	59.90	7.825	60.35	44.3	71	
	
Upper Extremity	18	37.11	7.894	36.00	24.5	58.2	
	
Physical Function	18	36.10	4.623	36.80	27.5	44.8	
	
Dyspnea	18	41.13	11.040	39.45	24.1	64.2	
