# Supplementary material for: Use of the patient-reported outcomes measurement information system (PROMIS®) to assess late-onset Pompe disease severity
Source: J Patient Rep Outcomes. 2020 Oct 9;4:83. doi: 10.1186/s41687-020-00245-2 (PMC7547055; doi:10.1186/s41687-020-00245-2)
Supplement: Supplementary file 2 — Additional file 2. [file 41687_2020_245_MOESM2_ESM.zip › T3_2_3_Average_T_score_Promis_gt_Median_PP6MWD.rtf]

Parameter	N	Mean	Standard
Deviation	Median	Min	Max	
	
Pain Interference	15	51.43	9.905	55.00	40.7	66.9	
	
Fatigue	15	57.37	8.308	56.60	44.3	77.8	
	
Upper Extremity	15	43.47	9.492	41.90	31	58.2	
	
Physical Function	15	39.26	7.164	37.60	27.5	57	
	
Dyspnea	15	37.31	7.780	34.80	27.3	52.1	
