# Supplementary material for: Use of the patient-reported outcomes measurement information system (PROMIS®) to assess late-onset Pompe disease severity
Source: J Patient Rep Outcomes. 2020 Oct 9;4:83. doi: 10.1186/s41687-020-00245-2 (PMC7547055; doi:10.1186/s41687-020-00245-2)
Supplement: Supplementary file 2 — Additional file 2. [file 41687_2020_245_MOESM2_ESM.zip › T3_2_4_Average_T_score_Promis_le_Median_PP6MWD.rtf]

Parameter	N	Mean	Standard
Deviation	Median	Min	Max	
	
Pain Interference	14	53.30	11.746	57.65	40.7	69.2	
	
Fatigue	14	55.96	11.688	54.10	33.1	71	
	
Upper Extremity	15	35.02	8.512	34.50	24.5	58.2	
	
Physical Function	15	37.11	8.670	36.80	26.2	62.7	
	
Dyspnea	15	42.09	12.096	40.70	24.1	64.2	
