# Supplementary material for: Use of the patient-reported outcomes measurement information system (PROMIS®) to assess late-onset Pompe disease severity
Source: J Patient Rep Outcomes. 2020 Oct 9;4:83. doi: 10.1186/s41687-020-00245-2 (PMC7547055; doi:10.1186/s41687-020-00245-2)
Supplement: Supplementary file 2 — Additional file 2. [file 41687_2020_245_MOESM2_ESM.zip › T3_2_Average_T_score_Promis.rtf]

Parameter	N	Mean	Standard
Deviation	Median	Min	Max	
	
Pain Interference	29	52.33	10.678	55.80	40.7	69.2	
	
Fatigue	29	56.69	9.922	55.60	33.1	77.8	
	
Upper Extremity	30	39.25	9.847	36.95	24.5	58.2	
	
Physical Function	30	38.18	7.891	37.00	26.2	62.7	
	
Dyspnea	30	39.70	10.285	38.60	24.1	64.2	
